# DRUM: A New Framework for Metabolic Modeling under Non-Balanced Growth. Application to the Carbon Metabolism of Unicellular Microalgae

**DOI:** 10.1371/journal.pone.0104499

**Published:** 2014-08-08

**Authors:** Caroline Baroukh, Rafael Muñoz-Tamayo, Jean-Philippe Steyer, Olivier Bernard

**Affiliations:** 1 INRA UR050, Laboratoire des Biotechnologies de l'Environnement, Narbonne, France; 2 INRIA-BIOCORE, Sophia-Antipolis, France; 3 LOV-UPMC-CNRS, UMR 7093, Villefranche-sur-mer, France; The George Washington University, United States of America

## Abstract

Metabolic modeling is a powerful tool to understand, predict and optimize bioprocesses, particularly when they imply intracellular molecules of interest. Unfortunately, the use of metabolic models for time varying metabolic fluxes is hampered by the lack of experimental data required to define and calibrate the kinetic reaction rates of the metabolic pathways. For this reason, metabolic models are often used under the balanced growth hypothesis. However, for some processes such as the photoautotrophic metabolism of microalgae, the balanced-growth assumption appears to be unreasonable because of the synchronization of their circadian cycle on the daily light. Yet, understanding microalgae metabolism is necessary to optimize the production yield of bioprocesses based on this microorganism, as for example production of third-generation biofuels. In this paper, we propose DRUM, a new dynamic metabolic modeling framework that handles the non-balanced growth condition and hence accumulation of intracellular metabolites. The first stage of the approach consists in splitting the metabolic network into sub-networks describing reactions which are spatially close, and which are assumed to satisfy balanced growth condition. The left metabolites interconnecting the sub-networks behave dynamically. Then, thanks to Elementary Flux Mode analysis, each sub-network is reduced to macroscopic reactions, for which simple kinetics are assumed. Finally, an Ordinary Differential Equation system is obtained to describe substrate consumption, biomass production, products excretion and accumulation of some internal metabolites. DRUM was applied to the accumulation of lipids and carbohydrates of the microalgae *Tisochrysis lutea* under day/night cycles. The resulting model describes accurately experimental data obtained in day/night conditions. It efficiently predicts the accumulation and consumption of lipids and carbohydrates.

## Introduction

Metabolic modeling is a powerful tool for bioprocesses to understand, predict and optimize the synthesis of intracellular molecules of interest [1]. The main interest of this approach relies on the use of the metabolic network knowledge and its associated stoichiometry. The kinetics modeling of each metabolic reaction is thus needed, especially to represent the transient dynamics of the set of intracellular compounds. However, the experimental difficulty to measure along time the dynamics of intracellular compounds hampers the modeling and calibration of the large set of reaction rates associated to the biochemical reactions of the metabolic network [2].

To overcome these hurdles, a commonly used hypothesis is the balanced-growth hypothesis, also called the Quasi-Steady-State Approximation (QSSA). Internal metabolites are assumed not to accumulate inside the microorganisms, which turns out to be a reasonable hypothesis for most of the microorganisms growing under constant conditions. This implies that every substrate uptake leads to microbial growth and products excretion. Thanks to this hypothesis, intracellular models are simplified and thus depend only on the stoichiometry of the network, the reaction reversibility and the uptake rate of the substrates.

Most of the metabolic modeling and analysis frameworks rely on the balanced-growth hypothesis. These frameworks include Flux Balance Analysis (FBA) [3], Dynamical Flux Balance Analysis (DFBA) [4], Elementary Flux Modes (EFM) [5], Flux Coupling Analysis (FCA) [6], Macroscopic Bioreaction Models (MBM) [7], Hybrid Cybernetic Models (HCM) [8] and Lumped Hybrid Cybernetic Models (L-HCM) [9]. Overall, these models predict well biomass growth and excreted products synthesis [4], [8], [10], [11] as long as the balanced-growth hypothesis is verified [12].

However, the balanced-growth hypothesis is unreasonable for microorganisms undergoing permanent environmental fluctuations. Indeed, in this case, the everlasting dynamics of intracellular accumulation and reuse play a key role in the cell metabolism. This is the case for phototrophic microalgae submitted to day/night cycles, which use photons to fix inorganic carbon during the day using photosynthesis. These promising organisms are seen as good candidates for production of third-generation biofuels thanks to their higher productivity compared to classical biofuels [13]. However, many improvements are necessary to become a cost effective and environmental-friendly bioprocess [14]. For that, a deep understanding of microalgae metabolism is necessary.

**Figure 1:**
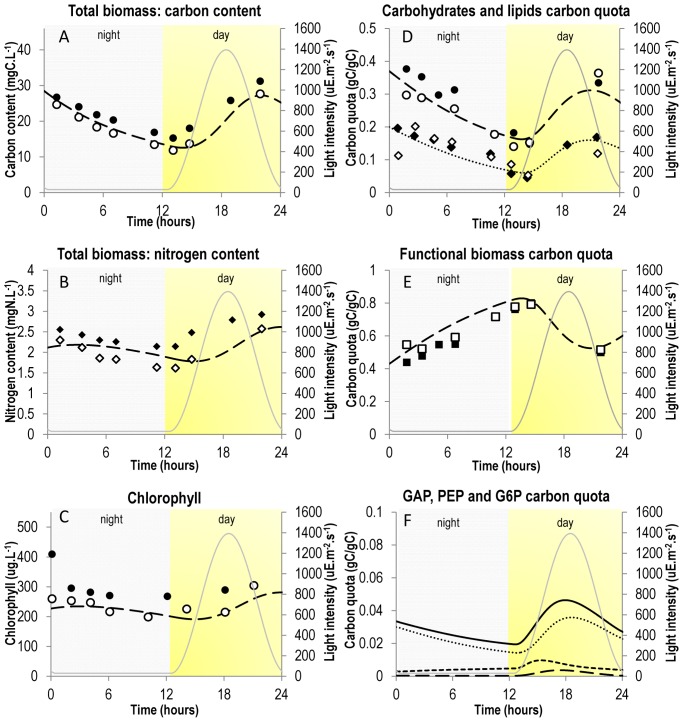
Comparison of simulation results with experimental data. Simulation results were obtained by simulation of system (7) and are represented by dashed or dotted lines. Experimental results were taken from [16] and are represented by dots, diamonds or squares. A. Evolution of total biomass in terms of carbon content. Dashed line: model; Circles: experimental data; Grey line: light intensity. B. Evolution of total biomass in terms of nitrogen content. Dashed line: model; Diamonds: experimental data; Grey line: light intensity. C. Evolution of chlorophyll (computed as a fixed percentage of functional biomass). Dashed line: model; Circles: experimental data; Grey line: light intensity. D. Evolution of “energy and carbon” metabolites. Dashed line and Circles: carbohydrates (CARB); Dotted line and Diamonds: lipids (PA); Grey line: light intensity. Accumulation of carbon and energy metabolites during the day and their consumption during the night for growth and maintenance purpose is well represented. E. Evolution of functional biomass *B*. Dashed line: model; Squares: experimental data; Grey line: light intensity. F. Evolution of “buffer” metabolites at branching points, as predicted by the model. Dashed line: glyceraldehyde 3-phosphate (GAP); Dotted line: glucose 6-phosphate (G6P); Small-dashed line: phosphoenolpyruvate (PEP); Black line: GAP + PEP + G6P; Grey line: light intensity. Note that their carbon mass quota is relatively small (less than 4%).

Microalgae store energy and carbon during the day so as to support growth and maintenance during the night, because of their autotrophic metabolism and the synchronization of their circadian cycle on the daily light [15]. Therefore, intermediate metabolites such as carbohydrates and lipids accumulate during the day and are remobilized during the night (Figure 1D) [16]. This behavior cannot be described under the balanced-growth assumption. One way to circumvent this issue is to represent these metabolites as product of the cell during the day and substrate during the night. Therefore applying one of the above-cited QSSA metabolic modeling frameworks could a priori be possible to represent carbon storage and better understand microalgae metabolism submitted to day/night cycles. In literature, only Knoop et al. [17], using the DFBA framework, computed metabolic fluxes for a full day/night cycle. However, determining an optimization function to represent carbon storage during the day and its consumption during the night is not a trivial task. Indeed, the classical optimization function “maximization of biomass production” does not work: when applying it, all the carbon available will go to biomass synthesis, and none to carbon storage. To circumvent this issue, the solution is to either force fluxes to carbon storage or to force the fluxes of biomass synthesis and maintenance (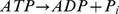) and other futile cycles. In their work, Knoop et al. [17], forced fluxes to carbon storage by changing the biomass composition at each time step. Their method indeed predicted metabolic fluxes dynamically but did not allow predicting the fluxes toward carbon storage and hence the dynamic change of biomass composition. In a context of better understanding and predicting microalgae metabolism for biofuels production, prediction of carbon storage fluxes is essential if one seeks the conditions in which microalgae accumulates more lipids or starch to improve biofuels production yield. Hence, to model such bioprocesses, a metabolic modeling framework that handles non balanced-growth and dynamics behaviors is necessary.

The aim of the present paper is to present DRUM (Dynamic Reduction of Unbalanced Metabolism), a new metabolic modeling framework, which allows to model dynamically intracellular processes where accumulation of metabolites plays a significant role. In a first section, the modeling approach and its mathematical translation are described. Then the approach is applied successfully to the carbon metabolic network of a unicellular microalgae (*Tisochrysis lutea*) in order to illustrate it on a realistic example, where simulation results are compared to experimental data. Finally, assumptions of the present approach and their implications are discussed in a last section along with the perspectives of the present work and the future possible applications.

## Method

Let us consider a continuous bioprocess implying microorganisms growing in a perfectly mixed stirred-tank reactor with constant volume, dilution rate *D* and incoming substrate *S_in_*. The microorganisms consume extracellular substrates represented by vector *S* to synthesize biomass *B* and produce excreted products represented by the vector *P*. The metabolic network of the microorganism is represented by the stoichiometric matrix 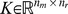 containing *n_m_* metabolites and *n_r_* reactions.

By applying a mass-balance, the bioprocess can be represented by the Ordinary Differential Equation (ODE) system:
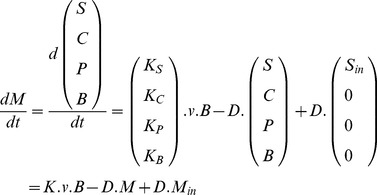
(1)where *M* represents the metabolites concentration vector composed of biomass *B*, uptaken substrates *S*, intracellular metabolites *C* and excreted products *P*. Concentrations are expressed in terms of solution concentrations, not concentrations per unit of cell. The kinetics vector 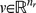 represents the reactions rates (per biomass unit) of the reactions of the metabolic network. By multiplication to *v*, biomass *B* acts as a catalyzer of kinetics *v*. Due to a lack of experimental data, *v* is often inferred [2]. The matrices 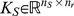, 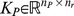, 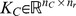 and 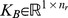 are the stoichiometric matrices of the metabolic network for the substrate, the products, the internal metabolites and the biomass (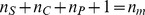). They are based on the knowledge of the metabolic network. The stoichiometric coefficients are thus known a priori, they do not need to be determined experimentally. The vector *M_in_* is the concentration vector of incoming metabolites in the chemostat, composed of incoming substrate *S_in_*.

**Figure 2:**
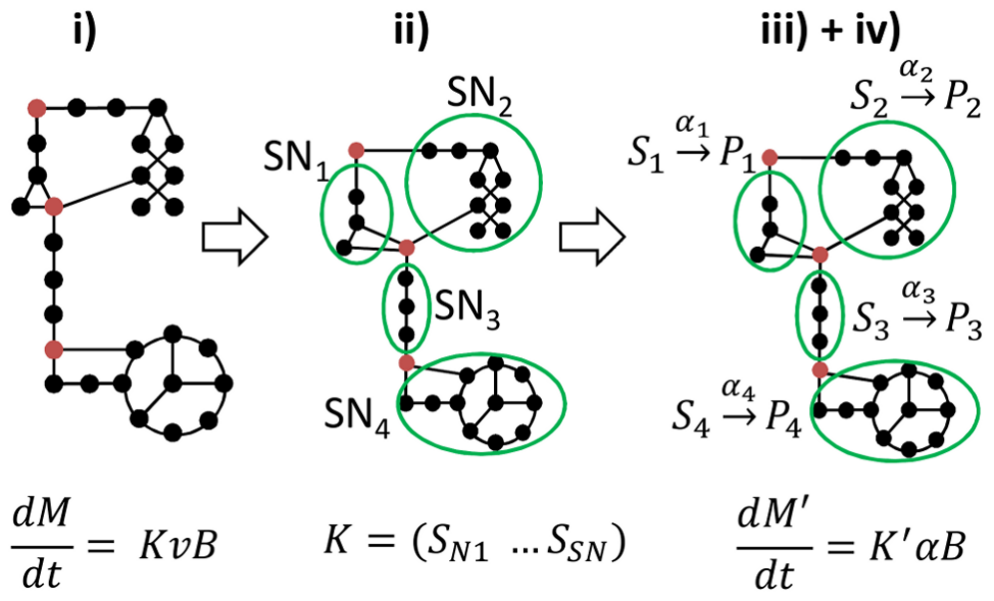
Modeling approach decomposed into 4 steps. The complete network (step i) is decomposed into sub-networks (SN) assumed at quasi-steady state (step ii). These are reduced to a set of macroscopic reactions (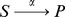) (step iii), for which kinetics are defined (step iv). Linking metabolites interconnecting the SN are allowed to accumulate (red circles) or be reused, which gives the dynamics of the whole network. From step iv), an ordinary differential equation (ODE) system is obtained, representing evolution of the macroscopic scale of the bioprocess as well as intracellular processes and accumulation of metabolites. In the full model described in step i), 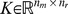,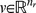, while for the resulting model provided by our approach, 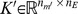 and 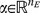, such that 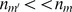 and 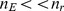.

The QSSA implies that internal metabolites do not accumulate (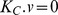). In the DRUM approach, instead, we assume that the QSSA is applicable only to groups of metabolic reactions that we call sub-networks (SNs). The remaining metabolites interconnecting the sub-networks, which we name *A* (*A* 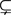 *C*), are not under the quasi-steady-state constraint. They are allowed to accumulate and thus can behave dynamically, which provides the dynamics to the whole network (Figure 2).

The QSSA for sub-networks relies on *i*) the presence of metabolic pathways corresponding to metabolic functions *ii*) the presence of group of reactions regulated together *iii*) the presence of different compartments in a cell (e.g., mitochondrion). Groups of reactions are thus determined taking into account these intracellular mechanisms. It is to be noted that some intracellular reactions can thus belong to several group of reactions. Mathematically, this is represented by redundant columns in the stoichiometric matrix *K*. The remaining metabolites (*A*) interconnecting the sub-networks formed using these rules are usually either situated at a branching point between several pathways or are end-products of metabolic pathways (e.g: macromolecules).

The sub-networks correspond mathematically to a partitioning of the stoichiometric matrix *K* into sub-matrices *K_SNi_* formed of grouped reactions: 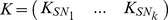
(2)where 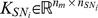 (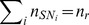) represents the sub-network *i* composed of i) incoming and outgoing metabolites *S_SNi_* and *P_SNi_* allowed to accumulate and ii) intermediate metabolites *C_SNi_* at quasi-steady state. *S_SNi_* and *P_SNi_* are either substrates S, products P, biomass B or intracellular metabolites A allowed to accumulate.

Each sub-network is assumed to be in a quasi-steady-state: 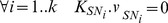
(3)


Under these assumptions and using elementary flux mode analysis [7], [12], [18], each sub-network can be reduced to a reduced set of macroscopic reactions:
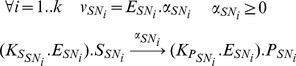
(4)where *E_SNi_* is the matrix of elementary flux modes of sub-network *SN_i_* and *α_SNi_* is the weight vector of the elementary flux modes. *α_SNi_* can be interpreted as the kinetics of the macroscopic reactions described by the stoichiometric matrix 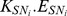 [12].

By grouping all the sub-networks, the following system is obtained:
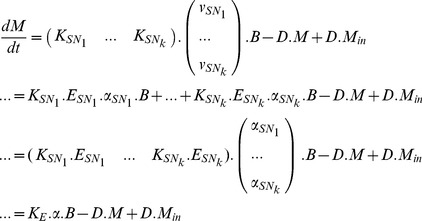
(5)


Only metabolites *A* are authorized to accumulate. Any other metabolite *C_j_*∈*C∖A* are assumed not to accumulate. Thus: 
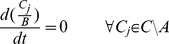
(6)


*C_j_*∈*C∖A* have simple dynamics. Hence a reduced dynamic model is obtained, defined by the metabolites vector 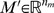 and the matrix 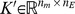, with *n_E_* the number of macroscopic reactions:
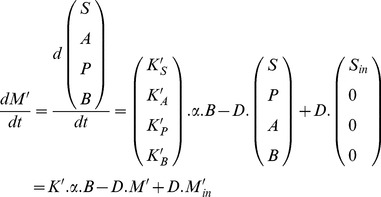
(7)


System (7) is a simplified version of (1) with the same structure but of much lower dimension, where accumulation of some internal metabolites (*A*) is allowed. Only the kinetics *α* of the resulting macroscopic reactions need to be determined. Classical kinetics found in literature are mass-action, power-law, Michaelis-Menten, Hill, cybernetic kinetics [19]. The choice is often arbitrary and the total number of parameters in the kinetics models needs to match the experimental data available so that a model validation is achievable. Once kinetics *α* are determined, all the metabolic fluxes can be computed using: 
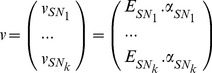
(8)


In the DRUM approach, particular attention has to be drawn to the definition of biomass *B*, which is no longer the conventional one. Biomass *B* is usually represented as an average composition of macromolecules present in the cell. With QSSA, any chemical element of substrate *S* ends up in either biomass *B* or excreted products *P*. But in the present approach, accumulation of internal metabolites is allowed. Hence, not all chemical elements from substrate *S* ends up in biomass *B* or products *P*; they can also be present in *A*. Total biomass (noted *X*) can then only be determined thanks to a mass-balance on each chemical element:
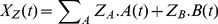
(9)where *Z* correspond to a chemical element (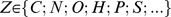), *Z_A_* and *Z_B_* corresponds to the number of chemical element *Z* per mole of accumulating metabolites *A* and biomass *B*, *A(t)* and *B(t)* correspond to the concentrations of *A* and *B* at time *t*, and *X_Z_(t)* correspond to the concentration of chemical element *Z* in total biomass *X* at time *t*.

To sum up, the DRUM approach is based on the following methodology, which is decomposed into a 4-step process (Figure 2):

- Find in the literature or build the metabolic network of the microorganism under study.
- Group metabolic reactions into sub-networks assumed to follow the QSSA.
- Reduce each sub-network to a set of macroscopic reaction using elementary modes analysis.
- Define kinetics for macroscopic reactions obtained and deduce an ODE system.

For sake of pedagogy, in the next section, the DRUM approach is illustrated on the carbon metabolism of unicellular microalgae.

## Results

### 1. Metabolic Network

To assess DRUM, experimental data of a continuous culture of *Isochrysis affinis galbana* (clone T-iso, CCAP 927/14) under day/night cycle was used [16]. This microalgae clone, known to accumulate high quantities of lipids was recently renamed *Tisochrysis lutea* [20]. Cultures were grown in duplicates in 5L cylindrical vessels at constant temperature (22°) and pH (8.2, maintained by automatic injection of CO_2_). The following measurements were performed: nitrates, particulate carbon and nitrogen, chlorophyll, total carbohydrates and neutral lipid concentrations [16].

With regards to the metabolic network, since *Tisochrysis lutea* has not been sequenced yet, no genome-scale metabolic network reconstruction was possible. Using the metabolic network of eukaryotic microalgae available (*Chlorella pyrenoidosa* [21], *Chlamydomonas reinhardtii* [22]–[27], *Ostreococcus tauri* and *Ostreococcus lucimarinus* [28]), we deduced a core carbon metabolic network common to unicellular photoautotrophic microalgae containing the central metabolic pathways (photosynthesis, glycolysis, pentose phosphate pathway, citric acid cycle, oxidative phosphorylation, chlorophyll, carbohydrates, amino acid and nucleotide synthesis). We did not represent species-specific pathways such as the synthesis of secondary metabolites since we assumed these pathways to have negligible fluxes compare to the main pathways and thus small impact on the other pathways. Indeed, secondary metabolites have very low biomass concentration compared to proteins, lipids, carbohydrates, DNA, RNA and chlorophyll. The reactions of synthesis of the macromolecules (proteins, lipids, DNA, RNA and biomass) were lumped, as classically done, into generic reactions where stoichiometric coefficients of the precursors metabolites were determined for *Tisochrysis* lutea thanks to their measured average quota in those macromolecules [16]. The detailed description of metabolic network reconstruction is available in File S1 section 1.

**Figure 3:**
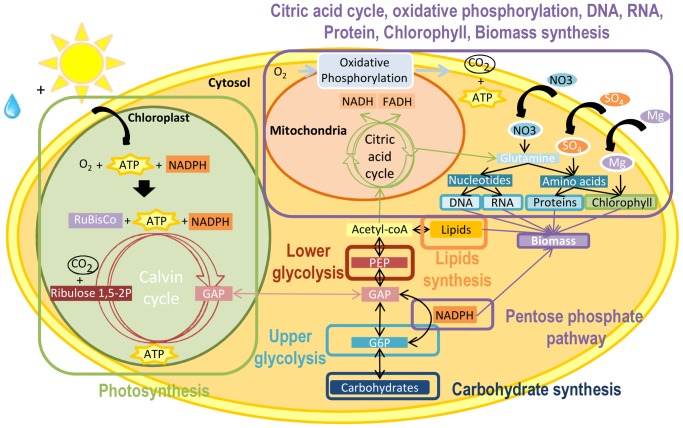
Simplified central carbon metabolic network of a unicellular photoautrotophic microalgae. Central carbon metabolic network is composed of photosynthesis in the chloroplast, transport reaction from the chloroplast to cytosol, glycolysis, carbohydrate synthesis, citric acid cycle, pentose phosphate pathway, lipids synthesis, oxidative phosphorylation, protein, DNA, RNA, chlorophyll and biomass synthesis. Photosynthesis is decomposed into two steps: the light step, which generates energy (ATP and NADPH) and oxygen using light and water and the dark step, which uses the generated energy to incorporate carbon dioxide. The end-product of photosynthesis is a 3 carbon sugar (here glyceraldehyde 3-phoshate written GAP), exported to the cytosol. GAP is situated in the center of glycolysis, and splits it into two parts: upper glycolysis and lower glycolysis. Upper glycolysis generates glucose 6-phosphate (G6P), which is then either invested for carbohydrates synthesis or in the pentose phosphate pathway to generate NADPH. Lower glycolysis generates phosphoenolpyruvate (PEP), which is then invested either in lipids synthesis or in the citric acid cycle, which produces necessary intermediate metabolites for proteins, DNA, RNA, chlorophyll and biomass synthesis. Cofactors (FADH, NADH) generated by citric acid cycle are transformed into energy (ATP) thanks to oxidative phosphorylation.

The resulting metabolic network is composed of the light and dark steps of photosynthesis in the chloroplast, the transport reaction from chloroplast to cytosol, glycolysis, carbohydrate synthesis, citric acid cycle, pentose phosphate pathway, lipids synthesis, oxidative phosphorylation, protein, DNA, RNA, chlorophyll and biomass synthesis (Figure 3). The network is composed of 157 internal metabolites and 162 reactions, including 13 exchange reactions with the environment and 1 internal exchange reaction (between the chloroplast and the cytosol). List of reactions and metabolites are available in File S1 section 2 and 3.

### 2. Formation and reduction of sub-networks

**Figure 4:**
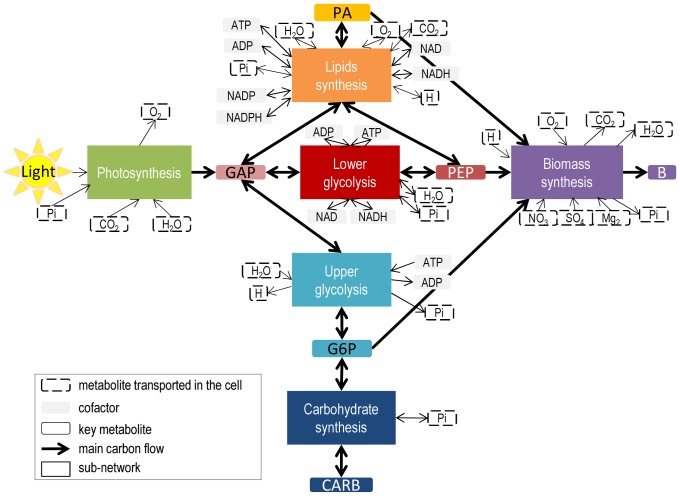
Central carbon metabolic network of a unicellular photoautrotophic microalgae decomposed into 6 sub-networks. The metabolic network was built by deducing a core carbon metabolic network common to unicellular photoautotrophic microalgae containing the central metabolic pathways of the metabolic network of eukaryotic microalgae available (*Chlorella pyrenoidosa* [21], *Chlamydomonas reinhardtii* [22]–[27], *Ostreococcus tauri* and *Ostreococcus lucimarinus* [28]) and experimental data of [16]. Details of the network reconstruction process and lists of reactions and metabolites are available in File S1 section 1–3. Metabolic reactions were grouped into sub-networks taking into account compartments and metabolic pathways. After reduction, 6 sub-networks were obtained corresponding to i) photosynthesis, ii) upper part of glycolysis iii) carbohydrate synthesis iv) lower part of glycolysis, v) lipids synthesis, vi) biomass synthesis. The resulting metabolites interconnecting the sub-networks and allowed to accumulate are either at branching points of metabolic pathways (glyceraldehyde 3-phosphate (GAP), glucose-6-phosphate (G6P) and phosphoenolpyruvate (PEP)) or end-products of metabolic pathways (lipids (PA), carbohydrates (CARB) and functional biomass (B)) or energy metabolites (ATP, ADP,NADH, NAD, NADPH, NADP) or metabolites transported in the cell (Light, CO_2_,O_2_,Pi,H_2_O,H,NO_3_,SO_4_,Mg). B corresponds to functional biomass and is composed of proteins, DNA, RNA, chlorophyll and lipids. List of macroscopic reactions for each sub-network is available in Table 1.

Metabolic reactions were grouped by metabolic functions, taking into account cell compartments and metabolic pathways. Six sub-networks were obtained (Figure 4) corresponding to *i*) photosynthesis, *ii*) upper part of glycolysis iii) carbohydrate synthesis *iv*) lower part of glycolysis, *v*) lipids synthesis, *vi*) biomass synthesis. Then, each sub-network was reduced to macroscopic reactions thanks to elementary flux mode analysis [18]. To compute elementary flux modes (EFMs) the software *efmtool* was used [29]. For all six sub-networks, the EFM could be computed easily, and their number was low (less than 30). It should be noted that an EFM analysis of the full network leads to 18776 modes (see File S1 section 4 for more details).

In the following sections, the formation and reduction of each sub-network is developed. The results are summarized in Table 1.

**Table 1:** Definition and reduction of sub-networks formed from metabolic reactions of a unicellular autotrophic microalgae.

Sub-network	Macroscopic reactions	Kinetics
Photosynthesis	30 Light + 3 CO_2_ + 2 H_2_O + P_i_ —> GAP + 3 O_2_ (MR1)	v_MR1_ = k_MR1_*I
Upper glycolysis	ATP + H_2_O —> ADP + P_i_ + H (MR2)	v_MR2_ = 0
	2 GAP + H_2_O —> G6P + P_i_ (MR3)	v_MR3_ = k_MR3_*GAP
	G6P + ATP —> H + ADP + 2 GAP (MR4)	v_MR4_ = k_MR4_*G6P
Lower glycolysis	GAP + ADP + P_i_ + NAD <—> PEP + ATP + NADH + H_2_O + H (MR5)	v_MR5_ = k_MR5_*GAP – k'_MR5_*PEP
Carbohydrate synthesis	G6P <—> CARB + P_i_ (MR6)	v_MR6_ = k_MR6_*G6P – k'_MR6_*CARB
Lipids synthesis	GAP + 16.61 PEP + 2 ADP + 13.46 NAD + 29.3 NADPH + 34.48 H + 2.15 O2 <—> PA + 14.61 Pi + 2 ATP + 13.46 NADH + 29.3 NADP + 4.31 H2O + 16.61 CO2 (MR7)	v_MR7_ = k_MR7_*PEP*GAP – k'_MR7_*PA
Biomass synthesis	3.13 PEP + 7.37 O_2_ + 4.46 H + 1.31 NO_3_ + 1.14 G6P + 0.11 PA + 0.03 SO_4_ + 0.0025 Mg —>B + 11.67 CO_2_ + 4.23 Pi + 6 H_2_O (MR8)	v_MR7_ = k_MR8_*PEP*G6P*NO_3_

Each sub-network was decomposed into a set of macroscopic reactions thanks to elementary flux mode analysis. List of reactions, incoming and outgoing metabolites for each sub-network are available in File S1 section 5. I corresponds to light intensity, expressed in µE.m-2.s-1.

#### 2.1 Photosynthesis

Photosynthesis allows phototrophic organisms to generate cell energy and incorporate carbon autotrophically. The process takes place in the chloroplast and is decomposed into two steps commonly called the light and dark steps. The light step consists in the generation of cell energy (ATP, NADPH) from water and photons, producing oxygen (R1). Thanks to the energy of the light step, the dark step incorporates carbon dioxide through Calvin cycle producing one 3 carbon sugar (3-phosphoglycerate written G3P). Then G3P is transformed in glyceraldehyde 3-phosphate (GAP) and transported to the cytosol of the cell (R14).

As both the dark and light step of photosynthesis takes place in the chloroplast and they both have the same metabolic function (to incorporate inorganic carbon), the reactions of the two steps were grouped into a sub-network and assumed at quasi-steady state. Elementary flux mode analysis yielded only one Elementary Flux Mode (EFM) (Table 1), giving one macroscopic reaction (MR1). The stoichiometry of the macroscopic reaction obtained is in agreement with literature: a quota of 10 photons are needed per carbon incorporated [24], [30].

#### 2.2 Upper glycolysis

As GAP is the end-product of photosynthesis and is situated at the center of glycolysis, glycolysis was split according to GAP into two sub-networks: lower glycolysis and upper glycolysis. In addition, dividing glycolysis into two parts is meaningful since upper glycolysis and lower glycolysis have different metabolic goals. Indeed, upper glycolysis synthesizes glucose 6-phosphate (G6P) to produce reductive power (NADPH) or to produce carbon storage compounds (carbohydrates), whereas lower glycolysis produces phosphenolpyruvate (PEP), which is then invested either in lipids synthesis or in the citric acid cycle to generate precursor metabolites for protein, DNA, RNA, chlorophyll and biomass synthesis.

G6P, instead of glucose, was chosen as the output of upper glycolysis because G6P is at a branching point between two metabolic pathways with different metabolic functions: carbon storage through the synthesis of carbohydrates and synthesis of NADPH reducing power through the pentose phosphate pathway.

Metabolic reactions of upper glycolysis were grouped and assumed at steady-state. Elementary flux mode analysis resulted in 3 macroscopic reactions (Table 1). Reaction (MR2) corresponds to a futile cycle since energy (ATP) is dissipated without creation of any metabolic product. This occurs when two metabolic pathways run simultaneously in opposite directions and have no overall effect other than to dissipate energy in the form of heat. Reaction (MR3) corresponds to G6P synthesis whereas reaction (MR4) corresponds to its consumption. The two equations cannot be compiled into one reversible reaction because of the irreversibility of the reactions transforming fructose 6-phosphate into fructose 1,6-biphosphate and vice-versa (R17-R18). Stoichiometry agrees with literature, since 1 ATP needs to be invested to transform 6-carbon sugars (G6P) into simpler ones (GAP) before getting 2 ATP back with lower glycolysis [31].

#### 2.3 Lower glycolysis

Lower glycolysis is a cascading set of reactions which generates the key metabolite phosphoenolpyruvate (PEP) and energy cofactors (ATP, NADH) from GAP. Lower glycolysis was cut at PEP instead of acetyl-coA (AcCoA) because of the presence of the anaplerotic reactions (R35, R36), converting oxaloacetate into PEP and vice-versa.

Lower glycolysis was assumed at steady state. One macroscopic reaction (MR5) was obtained with Elementary Flux Mode analysis (Table 1). Stoichiometry is in accordance with literature: after investment of one ATP in the upper part of glycolysis, 2 ATP are returned with one phosphoenolpyruvate [31].

#### 2.4 Carbohydrates synthesis

Carbohydrates (CARB) are complex sugars stored in the cell. They are formed from 6-carbon sugars (here G6P) by reverse glycolysis. All the reactions participating to carbohydrate synthesis were grouped and assumed to be in quasi-steady state. One reversible macroscopic reaction (MR6) was obtained by reduction thanks to elementary flux mode analysis (Table 1).

#### 2.5 Lipids synthesis

Lipids include a broad group of different macromolecules present in a cell. They contain at least one hydrophobic part and are constituted of long carbon chains linked to a sugar by an ether bound. In microalgae, only Triacylglycerols (TAGs) can be transformed into biofuels [32]. Unfortunately, lipid metabolism of microalgae is poorly known and it differs from bacteria and plants [33]. In the present network, lipids are represented by phosphatidic acids (PAs), precursors of many lipids including glycolipids and phospholipids for the membrane and TAGs for carbon storage.

All the reactions participating in lipids synthesis were grouped and assumed at quasi-steady state. One reversible macroscopic reaction (MR7) for the synthesis of PAs was obtained with elementary flux mode analysis (Table 1). Stoichiometric coefficients are non-integers because PAs are composed of two carbon chains with different lengths (C12–C20). To group all PAs under one entity, a generic reaction synthesizing an “average” PA (R123) was used. Its stoichiometric coefficients were determined experimentally using the proportion of the various fatty acids present in the cell (see File S1 section 1.1 for more details).

The macroscopic reaction obtained satisfies balance of the cofactors. For example 2 ADP yield 2 ATP, and 29.3 NADPH yield 29.3 NADP. Interestingly, when lipids are synthesized, some carbon atoms are lost through the production of CO_2_ and conversely some carbon atoms are gained when consuming lipids.

#### 2.6 Biomass synthesis

Protein, DNA, RNA and chlorophyll are necessary to synthesize biomass. Hence, all their synthesis reactions were grouped into a sub-network and assumed at quasi-steady state. Reactions for PA synthesis were not included because a dedicated sub-network is already present in the model. Therefore the biomass synthesis sub-network includes citric acid cycle, oxidative phosphorylation, pentose phosphate pathway, N and S assimilation, amino acids synthesis and nucleotide synthesis. Citric acid cycle takes place in the mitochondrion and transforms PEP into many precursor monomers for nitrogen assimilation, nucleotide and amino acids synthesis. For each run of the cycle, energy cofactors are generated (NADH, FADH2) and can be breathed into ATP thanks to oxidative phosphorylation. ATP is then reinvested into amino acids and nucleotide synthesis, necessary for DNA, RNA, protein and chlorophyll synthesis. Finally, reductive power (NADPH) necessary for nucleotide and amino acids synthesis is synthesized through the pentose phosphate pathway.

The reduction of this sub-network leads to 30 macroscopic reactions, in which 24 yields biomass (File S1 section 6). All macroscopic reactions not synthesizing biomass correspond to futile cycles where carbon is converted to energy, which is then dissipated. In terms of carbon, the 24 macroscopic reactions once normalized by unit of biomass synthesis flux were only different in their consumption of PEP and hence their production of CO_2_. A principal component analysis on the EFMs revealed that the difference was mainly due to two metabolic functions (incorporation of nitrogen and alanine synthesis) that could be performed following different pathways, some less energy-efficient than others explaining the difference of CO_2_ production (File S1 section 6, Figure S1, Figure S2).

We assumed that the cell was maximizing biomass growth, and hence minimizing carbon loss when synthesizing biomass. Therefore, the elementary flux mode normalized by unit of biomass synthesis flux with the best PEP/CO_2_ yield was chosen (Table 1). The resulting macroscopic reaction MR8 consumes PEP and NO_3_ for carbon and nitrogen sources, PA for functional and membrane lipids, G6P for NADPH synthesis through pentose phosphate pathway, SO_4_ and Mg for proteins and chlorophyll synthesis and O_2_ for ATP synthesis through oxidative phosphorylation. 42.4% of incoming carbon ends up in functional biomass; the rest is breathed through the TCA cycle because of energy demands met thanks to oxidative phosphorylation.

### 3. Macroscopic reaction kinetics and ODE system

**Figure 5:**
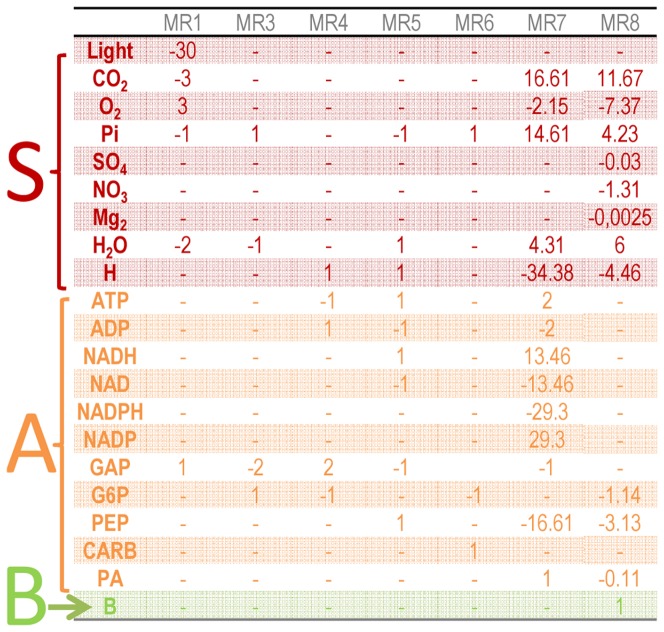
Stoichiometric matrix *K'* describing the bioprocess obtained after formation and reduction of metabolic sub-networks. *K'* as a much lower dimension (16×8) than the starting metabolic network (157×162). Lines of *K'* correspond to kept metabolites whereas columns correspond to macroscopic reactions obtained thanks to elementary flux mode analysis on each sub-networks. *K'* can be divided into sub-matrices *K_S_'* (in red), *K_A_'* (in orange) and *K_B_'* (in green), according to the lines corresponding to substrates *S*, intracellular metabolites allowed to accumulate *A* and functional biomass *B*.

After splitting the network into sub-networks and obtaining the EFMs for each sub-network, a reduced model described by 16 metabolites and 8 macroscopic reactions was obtained. The number of macroscopic reactions is similar to the model of Guest et al [34], where 10 lumped metabolic reactions were obtained. Mathematically, these first two steps of the DRUM approach translated into a reduced stoichiometric matrix *K'* (Figure 5) of much lower dimension (16×8) than the starting one (157×162). The definition of the reaction kinetics is the final building block of DRUM. For each macroscopic reaction obtained after the reduction step, simple proportional kinetics were assumed (Table 1).

According to section 2, the model is described by the following ODE system:
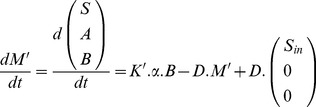
(10)where *M'* is the vector of kept metabolites (16×1) composed of substrate *S*, metabolites authorized to accumulate *A* and functional biomass *B*; *K'* is the reduced stoichiometric matrix (16×8) and α is the kinetics vector (8×1) (Figure 5 and Table 1).

As explained in section 2, biomass *B* corresponds to functional biomass. Total biomass, in terms of particulate carbon and nitrogen, is computed using the following formulae:
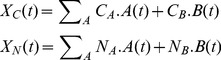
(11)where 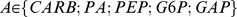, *C_A_* and *C_B_* correspond to the number of carbon atoms per molecule of *A* and *B*, *N_A_* and *N_B_* correspond to the number of nitrogen atom per molecule of *A* and *B*, *A(t)* and *B(t)* correspond to the concentration of *A* and *B* at time *t*, and *X_C_(t)* and *X_N_(t)* correspond to the concentration of carbon and nitrogen in total biomass *X*. As carbon and nitrogen biomass were measured experimentally, we simulated carbon and nitrogen content of the biomass. However, other chemical elements can be easily computed using the formula above. No additional parameters would be necessary as the above formula only uses chemical element composition and concentrations of *A* and *B*. Chemical element composition for *A* and *B* is available in section 1.5 of the File S1. In addition, energy cofactors are not taken into account in equation (11), as we assume their contribution negligible in terms of carbon and nitrogen compared to functional biomass and other molecules authorized to accumulate (CARB, PA, PEP, G6P & GAP).

Here, only the core metabolic network of a unicellular autotrophic microalgae was represented. It does not take into account energy necessary for mechanisms not represented by the network, like for instance the turnover of macromolecules and other so-called futile cycles. As it is clearly documented in the literature [35], energetic cofactors ATP, NADH, NADPH and FADH2 are difficult to balance. Usually, balancing is done through maintenance terms like equation MR2, which are determined so that growth rate and substrate consumption fits experimental data [24], [36]. Here, as carbon incorporation was not measured (light absorbed per unit of biomass was not measured, nor was CO_2_ dissolved concentration), estimation of maintenance and hence cofactors balance is difficult to perform. We thus decided not to consider the balance of energetic cofactors, and we did not describe their fate (ATP, ADP, NADPH, NADP, NADH, NAD).

The dynamic model has 10 degrees of freedom, each degree represented by a parameter that needs to be calibrated. To estimate parameters, we minimized the squared-error between simulation and experimental measurements (taken as an average of the duplicates) using the following formula:
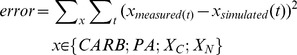
(12)


To minimize the error, the Nelder-Mead algorithm [37] (function *fminsearch* under Scilab (http://www.scilab.org)) was used. To reduce the risk of finding a local minima, several optimizations were performed with random initial parameters set. Then the set fitting the best experimental data was chosen. As very few data were available, all data were used to estimate model parameters. Results of parameter identification are presented in Table 2. The script file of the resulting model in Scilab format and the experimental data are available as File S2 and S3.

**Table 2:** Parameters obtained by the calibration of the model.

Parameters	Value
k_MR1_	11.07*10^−3^ µE^−1^.m^2^.s.mM.h^−1^.mMB^−1^
k_MR3_	223.53 h^−1^.mM B^−1^
k_MR4_	10.30 h^−1^.mM B^−1^
k_MR5_	436.95 h^−1^.mM B^−1^
k'_MR5_	5.00 h^−1^.mM B^−1^
k_MR6_	70. 00 h^−1^.mM B^−1^
k'_MR6_	6.50 h^−1^.mM B^−1^
k_MR7_	4.50 * 10^3^ mM^−1^.h^−1^.mM B^−1^
k'_MR7_	0.60 h^−1^.mM B^−1^
k_MR8_	2.18*10^4^ mM^−2^. h^−1^.mM B^−1^

### 4. Simulation

**Figure 6:**
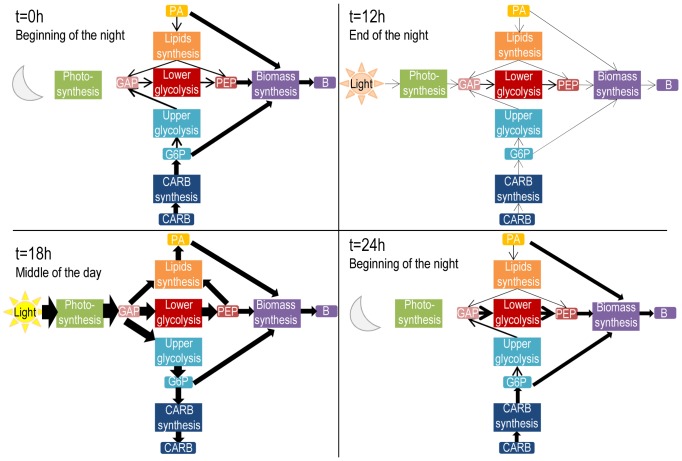
Fluxes between the 6 sub-networks at different time of the day. Fluxes were estimated thanks to model simulations. They were normalized per moles of carbon consumed or produced. Thickness of arrows depends on intensity of the flux. At the beginning of the night (t = 0 h), carbohydrates and lipids are already consumed so as to continue functional biomass growth. Most of carbohydrates and lipids are directly invested for biomass and only few of their carbons are used for PEP synthesis. At the end of the night (t = 12 h), the metabolism is slow, because very few carbons are left for growth and energy. At midday (t = 18 h), when light intensity is at its maximum, slightly less than a third of incoming carbons goes to functional biomass (28,6%). The rest of it is stored into carbohydrates (37,1%) and lipids (34,2%). After one day (t = 24 h), the biological systems has similar fluxes to the beginning (t = 0 h), showing the cyclic behavior of the metabolic network of a unicellular photoautotrophic microalgae submitted to a day/night cycle.

Model simulation reproduces accurately experimental data (see Figure 1). In particular, the model correctly represents lipids and carbohydrates accumulation during the day and their consumption during the night (Figure 1D). The distribution of fluxes during a classical day/night cycle is displayed in Figure 6 and in Video S1.

The model predicts a minimum of carbon storage (lipids and carbohydrates) one hour and a half after sunrise (13h37 and 13h17), when light intensity is sufficient to catch up with carbon loss through respiration. In a similar way, the maximum is reached three hours before sunset (20h50 and 21h02), when light intensity is insufficient to catch up with carbon loss through respiration (Figure 1D). Total carbon biomass follows a similar trend (minimum at 13h19 and maximum at 21h17), suggesting that an adequate harvesting time for biofuels production is three hours before sunset (21 h), when lipids are at their maximum. Interestingly, carbohydrates synthesis begins after and ends before lipids synthesis (respectively 13h31 and 22h08 against 12h58 and 23h26). This is due to the fact that there is a higher carbon demand for functional biomass synthesis from carbohydrates (through G6P) than from lipids: 6.84 carbons from carbohydrates are required per unit of functional biomass against 4.27 carbons from lipids. At midday (t = 18 h), when light intensity is at its maximum, carbohydrates and lipids synthesis are also at their maximum. At this time, slightly less than a third of incoming carbons goes to functional biomass (28.6%). The rest goes to carbohydrates (37.1%) and lipids (34.2%) storage (Figure 6).

Contrary to carbon storage, functional biomass carbon quota increases three hours before sunset until two hours after dawn, taking carbon from the lipids and carbohydrates pool (Figure 1D and E, Figure 6). Most of carbohydrates (through G6P) and most of lipids are directly consumed for functional biomass production. Only few of their carbons are used for PEP synthesis (Figure 6). At the end of the night and beginning of the day, the metabolism is really slow, because very few carbons in the storage pools are left for growth (Figure 6). Conversely, functional biomass carbon quota decreases during the day because of its dilution in the total biomass due to carbon storage. These obtained metabolic behavior are in agreement with the description of flux distribution given by Ross and Geider in [38].

Total biomass can be visualized in terms of particulate carbon and nitrogen (Figure 1A and B). Carbon follows a similar trend to carbohydrates and lipids, because carbon is only incorporated through photosynthesis during the day, and is lost during the night because of respiration to meet energy demands for continuing functional biomass growth. The diurnal photosynthetic quotient (moles of oxygen released per mole dioxide fixed) varies between 1.29 and 1.60 (Figure S3), depending on the light intensity, which agrees with the typical range of 1.0–1.8 for algae [22]. During the day, 79% of carbon loss is due to respiration and 21% to lipids synthesis. During the night, 10% of carbon lost by respiration is gained back by lipids consumption.

In the model, nitrogen content has exactly the same trend as functional biomass, since functional biomass is the only intracellular metabolite with nitrogen. It can be observed that there is slight delay in the uptake of nitrogen between the model and experimental data. In experimental data, the minimum is at sunrise and the maximum at sunset, meaning that *Tisochrysis lutea* stops incorporating nitrates as soon as the night starts. This time period corresponds to the period where cells divide [16]. Mocquet et al. in [39] have shown that nitrate uptake is stopped during cell division, which could explain the difference between predicted values and experimental data. However, including such mechanisms at this stage in the model would be debatable. Chlorophyll is also well predicted by the model, validating the hypothesis of a constant ratio with functional biomass.

Finally, it is interesting to look at the evolution of PEP, G6P and GAP concentrations predicted by the model. First, their concentrations are sufficiently low in terms of carbon, showing that carbon storage is mainly done with lipids and carbohydrates. However, their concentrations over time are not constant, and are particularly different between day and night. Indeed, their concentrations are much higher during the day than during the night, giving certain flexibility to the metabolic network when environmental conditions changes rapidly (here light). The ability of metabolic network to face permanent fluctuating environmental conditions consolidates one of the advantages of the DRUM approach. Such flexibility is acquired through certain metabolites, which can accumulate and therefore act as buffers. This could not be achieved with a steady-state assumption.

## Discussion

### 1. Assumptions in the DRUM approach

#### 1.1 QSSA on sub-networks

The main assumption of the DRUM approach is the quasi-steady state assumption on sub-networks of the metabolic network. This assumption is supported by the idea of cell function and cell compartment, often associated to co-regulation and substrate channeling.

Indeed, in a cell, metabolic pathways composed of grouped reactions regulated together are omnipresent. These reactions are often synchronous: intermediate metabolites produced by a reaction are nearly immediately consumed by the next reaction in the cascade. This implies a quasi-steady state for the intermediate metabolites. Many examples of such pathways can be found in literature. One of the most illustrative ones is reactions in cascade where the first reaction of the pathway is submitted to feedback inhibition by the end-product of the last reaction [40].

In addition, spatial and molecule crowding are not negligible phenomena in a cell. When not taken into account, they imply that any intracellular metabolite can be consumed in any reactions of the cell, even if the reaction occurs at a far loci or in a different compartment where the molecule cannot be transported to and needs to be resynthesized. This often leads to erroneous metabolic flux distributions when using flux balance analysis and to a combinatorial explosion of the number elementary flux modes representing the metabolic network. For example, in the case of the metabolic network of *Chlamydomonas reinhardtii* [24], when ATP of the chloroplast is constrained to stay in the chloroplast, the number of EFMs reduces from 4909 to 452. We thought reasonable to assume that reactions inside a same compartment and completing the same metabolic function are synchronous. For example, the light and dark steps of photosynthesis can be assumed synchronized so that all ATP and NADPH produced by the first step are directly consumed in the second step.

An extreme illustration of space phenomena supporting our quasi-state assumption is substrate channeling, where an intermediate metabolite is, instead of being released in the solution, passed from enzyme to enzyme so as to avoid any loss to competing pathways [41]. In this case, the notion of metabolic reaction is difficult to define since the reaction is already a macroscopic reaction composed of synchronous elementary reactions where intermediate metabolites are under QSSA.

Even if regulation, substrate channeling and reactions loci in the cell are not always well-known, we assumed that QSSA is a biologically reasonable assumption for a group of reactions taking place in the same compartment, synthesizing a same pathway end-product or fulfilling a similar metabolic function. QSSA on sub-network is a mild way to relax the balanced growth hypothesis, without constraining the full network anymore. In most cases, the main sub-networks will be the same, defined on metabolic functions: upper glycolysis, lower glycolysis, TCA cycle, Calvin cycle (for photoautotrophs), macromolecules synthesis.

It is very important to keep in mind that the DRUM approach does not only split the initial network into sub-networks, but it also duplicates some reactions that take place simultaneously at different part of the cell within different functions. This point is very important in order to keep a sound meaning to the reduced networks derived from the EFM analysis.

#### 1.2 Network splitting into groups of reactions

Network splitting into groups of reactions is performed on the basis of the above-mentioned criteria. However, these intracellular mechanisms are not always well known. Hence, it is difficult to split the network only taking into account experimentally proved report of these phenomena on the microorganism studied. To overcome this hurdle, network splitting was also performed thanks to educated guesses using the topology of the metabolic network, the known metabolic functions of some groups of reactions, the experimentally known accumulating metabolites (e.g., lipids, carbohydrates) and the key topological place of some metabolites. The metabolites *A* allowed to accumulate are thus end-products of metabolic pathways (e.g., macromolecules) or situated at a branching point between several pathways.

In the case of *Tisochrysis Lutea*, the presence of the chloroplast compartment was used to assume QSSA for photosynthesis. For the rest of the metabolic network, reactions were grouped according to known metabolic functions: carbohydrate synthesis, upper glycolysis, lower glycolysis, lipids synthesis, biomass synthesis. The accumulated metabolites GAP, PEP, G6P were chosen because situated at branching points of several metabolic pathways. Indeed, GAP, the output of photosynthesis, is situated at the middle of glycolysis and is also an output of the pentose phosphate pathway. G6P is situated at the branching point between carbohydrates synthesis and the pentose phosphate pathway. Finally, PEP is situated at the branching point between lipids synthesis, the TCA cycle for precursor metabolites necessary for biomass synthesis and the anaplerotic reactions.

However, the choice of the decomposition is not totally straightforward. The splitting of *Tisochrysis lutea* metabolic network was performed by trial and errors with different possible decompositions. Several possible configurations were tested and the best one was kept. For example, the metabolic network was cut, instead of glyceraldehyde 3-phosphate (GAP) at glycerone-phosphate (DHAP) and instead of phosphoenolpyruvate (PEP) at pyruvate (PYR). To cut at PEP seemed a better choice to fit functional biomass data, but cutting at DHAP did not influence the results since DHAP and GAP are interchangeable metabolites (‘DHAP <–> GAP’ (EC 5.3.1.1)). Whether the network should be cut at GAP or DHAP could only be answered with additional experimental measurements.

In a general way, only few decompositions work, but some have close performances. Only experimental data will allow favoring one from the other. Still, the presence of these equivalent decompositions is beneficial since it points out the dynamic measurement of metabolites to make so as to discriminate the best model.

The method, in its first developmental stage, is not automatic yet. However, systematic network splitting techniques could be developed. For example, the network could be split according to the metabolites participating in more than a threshold number of metabolic reactions [42]. The network could also be split using flux coupling analysis, where totally coupled reactions could be used as a starting point for sub-networks [43]. Finally, any other network clustering techniques could be used, from metabolic function annotations to topology [44], [45]. In addition, automation of the method will allow discriminating the different possible decompositions. Indeed, the automated decomposition algorithm will yield a finite number of possibilities, which will be explored. For each of them, a finite number of simple kinetics will be tested and their kinetic parameters estimated to fit experimental data. The Akaike Information Criterion could then be used to provide a score for selecting the best candidate model [46], accounting for the tradeoff between fitting and parameter parsimony. However, selecting the best decomposition imposes a computational challenge since global identification procedures, often requires, in practice, expert knowledge to reduce the attraction of local minima.

#### 1.3 Network reduction into macroscopic reactions

Once network splitting into sub-networks was performed, network reduction is straightforward as it consists in computing Elementary Flux Modes (EFMs) for each sub-network and reducing them to macroscopic reactions by keeping only the transport reaction of incoming and outgoing metabolites. This can be performed automatically using softwares like *efmtool* [29] to compute the EFMs and a small script to deduce the macroscopic reactions from the EFMs obtained.

However there is an exponential explosion of the number of Elementary Flux Modes (EFMs) when the number of reactions increases, which implies an exponential explosion of the kinetics parameters to estimate. This could make the approach intractable and annihilate the advantage of DRUM compared to a full kinetics model when using large sub-networks resulting for example from the splitting of a genome-scale metabolic network. To overcome this difficulty, small sub-networks should be favored and there are available methods to reduce the number of EFMs such as the use of experimental data [7], a projection of the EFMs space into the yield space [47] or the clustering of EFMs into phenotypic families [48]. These methods are semi-automatic, well documented and already proved to be efficient to model biological systems [7], [11], [49]. Flux Balance Analysis (FBA) and by extent Dynamic Flux Balance Analysis (DFBA) can also be seen as methods to reduce the number of EFMs using optimization. Indeed, a solution of FBA corresponds to a positive linear combination of EFMs and the solution for any optimal product/substrate ratio always coincide with an elementary mode [5]. Thus, when applying DRUM, such above-mentioned methods can be automatically applied if the number of EFMs for some sub-network is too high.

In the case of *Tisochyris lutea*, the biomass synthesis sub-network is composed of 105 reactions. The calculation of the EFMs resulted in 24 macroscopic reactions. Note that the number of macroscopic reactions is already lower than the number of reactions of the original sub-network. For a further reduction, we kept the EFM with best PEP/CO_2_ yield when normalized by unit of biomass synthesis flux, which was the same as optimizing biomass growth since we minimized carbon loss through oxidative phosphorylation.

In addition, DRUM drastically reduces the number of EFM compared to a QSSA applied to the whole network thanks to the application of QSSA only on sub-networks. Indeed, as EFMs are only computed on small sub-networks and as the explosion of the number of EFMs is exponential with the number of reactions, the sum of the number of EFMs obtained from each sub-network is smaller than the number of EFMs obtained for a QSSA on the whole network. In the case of *Tisochrysis lutea*, DRUM reduces the number of EFM from 18776 for the whole network down to 11. This implies a low number of degrees of freedom (10 parameters) compared to the other methods (cf Table 3) where degrees of freedom are often hidden in parameters (e.g.: biomass composition) or imposed fluxes (substrate consumption, product formation, biomass growth, maintenance) varying along discrete time instants.

**Table 3:** Comparison of existing microalgae models representing carbon storage.

Reference	Modeling type	Macroscopic reactions	Metabolic Fluxes	Metabolites concentrations	Degrees of freedom
[34]	Macroscopic, Dynamic	11	0	7	12
[38]	Macroscopic, Dynamic	5	0	7	18
[53]	Macroscopic, Dynamic	3	0	4	12
[54]	Macroscopic, Dynamic	6	0	7	9
[55]	Macroscopic, Dynamic	4	0	5	15
[56]	Macroscopic, Dynamic	1	0	2	5
[57]	Macroscopic, Dynamic	2	0	3	7
[58]	Macroscopic, Dynamic	11	0	7	8
[59]	Macroscopic, Dynamic	6	0	7	7
[24]	Metabolic, Static	0	160	0	1
[22]	Metabolic, Static	0	484	0	2
[26] & [52]	Metabolic, Static	0	280	7	22
[17]	Metabolic, Static & Dynamic	0	760	9	45
Present approach	Metabolic & Macroscopic, Dynamic	7	162	14	10

To compare the models, our definition of “degrees of freedom” stands for the number of information needed to simulate the models. For macroscopic models, degrees of freedom relate to the kinetic parameters of the model. For FBA models, degrees of freedom relate to the number of constraints needed to determine the flux distribution. Incoming light and biomass composition were not considered as degrees of freedom.

For [56] and [57], no macroscopic reactions are obtained per se, as growth is independent of nutrient uptake. Only population growth is represented (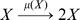).

For [17], 7 biomass compositions were necessary to perform DFBA. We counted 6 of them as degrees of freedom.

#### 1.4 Macroscopic reactions and their kinetics

Once all macroscopic reactions modes are obtained, their kinetics need to be defined, which is the final step of DRUM. This is a delicate task, and unfortunately there is no unique or systematic way of doing it. The choice is left to the researcher's attention and experience and is also relative to the experimental data available. Classical kinetics found in literature are mass-action, power-law, Michaelis-Menten, Hill, cybernetic kinetics [19], or more complex allosteric regulations kinetics [50]. However, DRUM is an approach looking for a model with a reduced complexity and hence a minimum number of parameters.

In the case of *Tisochrysis Lutea*, since one parameter per reaction turns out to be sufficient to explain the data, we kept this minimum structure to follow a parsimony principle.

In future works, methods such as the one developed by *Curien et al.* [50], based on in vitro reconstitution of the sub network, could provide a way to experimentally determine kinetic models. Alternatively, a multi-level optimization such as in [51] could also be used. It would avoid the need to postulate kinetics and estimate their parameters. Yet, defining the objective function is not a trivial task.

#### 1.5 Total biomass and functional biomass

Biomass *B* is a variable used to predict the macroscopic biomass production, which is generally measured in dry weight mass or in carbon mass. In metabolic models, biomass *B* is usually represented as an average composition of macromolecules present in the cell. For example, in the case of *Chlamydomonas reinhardtii*, the biomass is composed of 64.17% of proteins, 27.13% of carbohydrates, 4.53% of lipids, 3.05% of RNA, 1.02% of chlorophyll and 0.11% of DNA in average [24]. An artificial metabolic reaction of biomass synthesis is thus added to the metabolic network, where the stoichiometric coefficients of the reaction are the measured molar proportions of each macromolecule present in the cell. In system (1), biomass *B* acts as a growth catalyzer. This reflects the fact that the proteins, nucleic acids and other macromolecules that are part of the biosynthetic apparatus and structural material (e.g., cell walls) catalyze the intracellular reactions and hence growth.

In the DRUM approach, some macromolecules can accumulate and will therefore not appear in biomass *B*. We assumed that macromolecules catalyzing growth such as proteins do not accumulate and end up in biomass *B*, which we rename functional biomass *B*. This relies on the assumption that storage compounds of a cell does not have any other metabolic functions than to store chemical elements (e.g., carbon) so as to supply energy and chemical elements demands to continue growth when these resources are no longer available in the environment. The term α*B* in (7) is thus still meaningful, since functional biomass *B* catalyzes growth as the term *vB* does in (1). An estimation of the total actual biomass can then be obtained by summing up functional biomass *B* and the storage terms *A* (cf equation (9)).

### 2. Comparison to other models

Microalgae models exist for more than 60 years and can be divided into two main categories: dynamical macroscopic models (see [15] for a full review) and static metabolic models [17], [22], [24], [26], [52].

To date, there is only 9 macroscopic models representing carbon storage (particularly lipids) in microalgae [34], [38], [53]–[59]. However, these models are empirical and do not rely on metabolic knowledge. They describe efficiently some key metabolites, but does not allow to understand the intracellular mechanisms taking place in the cell and stay limited in the number of variables for which accumulation dynamics can be forecasted (Table 3). Only the models of [34] and [58] tried to incorporate some metabolic knowledge. Guest et al [34] used lumped metabolic reactions taken from literature and for which stoichiometric coefficients were determined depending on the environmental conditions. Fleck-Schneider et al [58] used a hybrid modeling technique where ordinary differential equations described the macroscopic scale of the bioprocess whereas flux optimization on a lumped metabolic model was performed at each time-step at the metabolic scale.

For metabolic models, only static flux predictions under constant light were made, where lipids and carbohydrates were at a constant ratio in biomass [17], [22], [24], [26], [52]. Even if, sometimes, the influence of light intensity on metabolic fluxes and biomass composition was studied [24], [52], only the recent model of Knoop et al [17] tried to simulate, thanks to dynamic flux balance analysis, the evolution of metabolic fluxes during a day/night cycle. The simulation was performed thanks to a time-dependent biomass reaction based on literature, which allowed forcing the value of the fluxes to the storage compounds. This involves a much higher degree of freedom (45, cf Table 3) than with DRUM (10) since the biomass composition must be postulated at each time instant (or at some key instants and then interpolated). However, a more systematic method for representing carbon accumulation and consumption over time is lacking. Contrary to the work of Knoop and al. [10], DRUM allows predicting at the same time all metabolic fluxes and the change of biomass composition without forcing carbon storage to a given value computed at each time step. This is the real advantage of our method, where we can predict at the same time the macroscopic scale (biomass synthesis, substrate consumption, and products synthesis) and the intracellular scale (metabolic fluxes). To the authors' knowledge, no one managed to predict them dynamically using a metabolic framework managing non-balanced growth.

In relation to the existing microalgae models DRUM, the new framework proposed in this paper, allowed for the first time to predict dynamically at the same time the macroscopic scale of the bioprocess (particulate carbon and nitrogen, Figure 1A and B) and the metabolic scale (lipids, carbohydrates, chlorophyll and all metabolic fluxes, see Figure 1C and D and E, Figure S4) with few parameters to estimate (Table 3). The originality of DRUM lies in the coupling of macroscopic and intracellular modeling approaches as discussed below.

### 3. Joining the macroscopic and the metabolic scales: a bottom-up approach

Classical modeling approaches of bioprocesses can be sorted into two main categories: modeling at the macroscopic scale, where microorganisms act as catalyzers of macroscopic reactions [60] and modeling at the intracellular scale, which takes into account intracellular mechanisms such as biochemical reactions or genetic regulation.

Macroscopic models have usually a low dimension, allow to account for time varying experimental data and predict well the macroscopic scale of bioprocesses such as substrate consumption and biomass growth [60]. Unfortunately, the number of macroscopic reactions necessary to represent the bioprocess, their expression, their stoichiometric coefficients and their kinetics need to be determined experimentally [61], [62]. In addition, macroscopic modeling does not take into account intracellular mechanisms and thus can hardly be used for optimization of intracellular molecules of interest.

On the other hand, intracellular modeling describes accurately mechanisms occurring inside the cell such as reactions between metabolites catalyzed by enzymes, translation and transcription of genes. These models are based on the knowledge of the metabolic, transcriptomic and genomic networks. They allow a better understanding of the cellular mechanisms and seem more appropriate to describe and optimize bioprocesses implying intracellular molecules. However, the use of intracellular models for time varying experiments is hampered by the lack of experimental data required to define and calibrate the kinetic reaction rates of the biochemical reactions [2]. The common assumption found in the literature to overcome this hurdle is the balanced-growth assumption.

While these two modeling approaches bring answers to different objectives, a remaining challenging question is how to couple macroscopic and intracellular models to enlarge the prediction capabilities of the model while keeping a model structure with a low complexity level?

Two strategies can be applied in the attempt to couple the two scales: a top-down approach, where some intracellular mechanisms are included in details in a macroscopic model, or a bottom-up approach where intracellular mechanisms are simplified and linked to the macroscopic scale. The first approach consists in finding and representing in details the preponderant intracellular mechanisms that have an impact at the macroscopic scale. All others intracellular mechanisms are assumed negligible. This approach is thus very microorganism dependent and cannot easily be generalized. Still, even if limited, this approach usually improves the prediction of the macroscopic scale and helps to better understand the bioprocess [38], [63].

On the other hand, the reduction of intracellular mechanisms to represent in a simple way the macroscopic scale of a bioprocess is a difficult task, particularly given the lack of knowledge of intracellular mechanisms and the lack of experimental data available. Still, thanks to the balanced-growth hypothesis, systematic reduction frameworks were already developed for the metabolic scale. Indeed, QSSA allows to link statically [3] or dynamically [7], [9] the intracellular scale (metabolic fluxes) to the macroscopic scale (biomass growth). Even if some difficulties still remain (e.g., a high number of elementary flux modes, no accumulation of intracellular metabolites, balance of cofactors), predictions are in good agreement with experimental data and allow insightful understanding and optimization of bioprocesses [7], [9], [11]. DRUM is the next generation of these existing bottom-up approaches, where dynamics and intracellular accumulation are taken into account, as well as spatial phenomena and regulation to some extent, thanks to the network splitting.

### 4. Use of DRUM to guide metabolic engineering

Gene deletion studies (GDS) exploit the Gene-Enzyme-Reaction relationship to predict the effect of the deletion of one or several genes on the growth and/or on product synthesis [64]–[68]. Metabolic engineering can thus be guided thanks to *in silico* models by GDS to find ideal gene targets to improve production yields of molecules of interest. The DRUM approach could extend these approaches at the levels of the metabolic function or of the reaction.

The first level consists in targeting metabolic functions represented by the macroscopic reactions deduced from the EFMs of each sub-networks. Deleting a metabolic function is hence equivalent to delete a macroscopic reaction. In a practical way, as EFMs are minimal metabolic behaviors of the cell [69], targeting an EFM is the same as targeting one of the EFM non-null reactions, since EFMs are non-decomposable vectors by definition [69]. However one needs to be careful that the deletion of one reaction does not affect another EFM using the same reaction.

The second level is the deletion of a reaction in the metabolic network. This could yield the same result as deleting one metabolic function, yet it could also imply accumulation of a previously non-accumulating metabolite hence modifying the decomposition of the sub-networks. It could also imply obtaining different EFMs and hence different macroscopic reactions (e.g.: stoichiometric coefficients). This could require a new decomposition and reduction of the sub-networks, and new kinetics to postulate and parameters to estimate.

For *Tisochrysis* lutea, the goal of our microalgae model was to better apprehend the carbon metabolism of microalgae in day/night cycles. It is clear that such a model has many direct implications for metabolic engineering with microalgae. The fact that cells can store very high amounts of lipids with a daily pattern has clear consequences on the harvesting period (section 3.4). It also indicates the paths and the enzymes to be targeted in order to more efficiently accumulate lipids. For example, we can target the carbohydrates production (MR6) and simulate *de novo* the model to see whether it has an impact on lipids accumulation. The results suggests, as expected, that the carbohydrates storage pool diminished quickly at the expense of the lipids and functional biomass pool (Figure S5, File S1 section 7). In addition, G6P accumulates during the day and is consumed during the night, standing in for the carbohydrates storage pool. The only difference is that at the end of the night, the G6P pool is completely depleted. What is also interesting is that the total carbon biomass *X* stays the same: only a shift of carbon between the different pools is observed. The day/night cycle growth still occurs and takes place at a similar velocity, which was not straightforward since glucose-6-phosphate concentration could have been too low to allow functional biomass synthesis during the night.

## Conclusions

This paper presents DRUM, a new metabolic modeling framework, which allows to predict dynamically the accumulation of intracellular metabolites using metabolic knowledge. The proposed strategy results from a tradeoff between complexity and representativeness. It conciliates intracellular and macroscopic models in a fluctuating environment.

DRUM was applied to the phototrophic unicellular microalgae *Tisochrysis lutea* and led to a model describing well the accumulation of lipids and carbohydrates in the microalgae under day/night cycles.

DRUM helps to better understand intracellular mechanisms at the metabolic level when the biological system undergoes environmental perturbations. In addition, DRUM could be used in dynamic control frameworks to optimize the bioprocess. This was not possible before, as models were static and did not allow accumulation of intracellular metabolites.

Future work will consist in applying the methodology to mixed ecosystems, so as to better understand the interactions taking place between the individual species composing the microbial community. Indeed, even if the scale is different, same philosophical principles can be used to split the metabolic network of a microbial community.

## Supporting Information

Figure S1 **Projection of elementary flux modes obtained from the biomass synthesis sub-network in the PEP/CO2 yield space.** The reduction of the biomass synthesis sub-network leads to 30 macroscopic reactions, in which 24 yields biomass. In terms of carbon, the 24 macroscopic reactions were only different in their consumption of PEP and hence their production of CO_2_. A projection in the yield space PEP = f(CO_2_) reveals two distinct metabolic behaviors.
(TIF)

Figure S2 **Principal component analysis of the elementary flux modes obtained from the biomass synthesis sub-network.** The difference in the PEP/CO2 yield is mainly due to two metabolic functions (incorporation of nitrogen (x-axis) and alanine synthesis (y-axis)) that can be performed thanks to different pathways, some less energy-efficient than others explaining the difference in CO_2_ production.
(TIF)

Figure S3 **Predicted photosynthetic quotient during a day/night cycle.** The quotient varies between 1.29 and 1.60, depending on the light intensity, which agrees with the typical range of 1.0–1.8 for algae [22].
(TIF)

Figure S4 **Metabolic fluxes of the core network at midday (18 h).**
(PNG)

Figure S5 **Comparison of the wild type and MR6-deficient** ***in silico*** **models.** The two models were then simulated for 48 h, one with k_carb_ = 0 h^−1^.mM B^−1^, the other one with k_carb_ = 70.00 h^−1^.mM B^−1^. The dilution rate and the incoming substrate concentrations were set at 1 days^−1^ and 4.018 mgN.L^−1^.
(PNG)

File S1 **Detailed metabolic network reconstruction process of** ***Tisochrysis lutea*** **; list of reactions and metabolites; analysis of the whole metabolic network; list of sub-networks; list of macroscopic reactions obtained for the biomass synthesis sub-network.**
(PDF)

File S2 **Scilab script of the day/night cycle model of** ***Tisochrysis Lutea*** **.**
(SCE)

File S3 **Experimental data of continuous cultures of** ***Tisochrysis Lutea*** **.**
(XLS)

Video S1 **Predicted metabolic fluxes between sub-networks during a 24 h day/night cycle.**
(MP4)

## References

[1] Stephanopoulos G, Aristidou AA, Nielsen J (1998) Metabolic engineering: principles and methodologies. 1st ed. San Diego: Academic Press - Elsevier USA.

[2] Heijnen JJ, Verheijen PJT (2013) Parameter identification of in vivo kinetic models: Limitations and challenges. Biotechnol J 8: 768–775. 23813763 10.1002/biot.201300105

[3] Orth J, Thiele I, Palsson B (2010) What is flux balance analysis? Nat Biotechnol 28: 245–248. 20212490 10.1038/nbt.1614 PMC3108565

[4] Mahadevan R, Edwards JS, Doyle FJ (2002) Dynamic flux balance analysis of diauxic growth in Escherichia coli. Biophys J 83: 1331–1340. 12202358 10.1016/S0006-3495(02)73903-9 PMC1302231

[5] Schuster S, Dandekar T, Fell DA (1999) Detection of elementary flux modes in biochemical networks: a promising tool for pathway analysis and metabolic engineering. Trends Biotechnol 17: 53–60. 10087604 10.1016/s0167-7799(98)01290-6

[6] Burgard AP, Nikolaev V, Schilling CH, Maranas CD (2004) Flux coupling analysis of genome-scale metabolic network reconstructions. Genome Res 14: 301–312. 14718379 10.1101/gr.1926504 PMC327106

[7] Provost A, Bastin G, Agathos SN, Schneider Y-J (2006) Metabolic design of macroscopic bioreaction models: application to Chinese hamster ovary cells. Bioprocess Biosyst Eng 29: 349–366. 17013615 10.1007/s00449-006-0083-y PMC1764600

[8] Song H-S, Morgan JA, Ramkrishna D (2009) Systematic development of hybrid cybernetic models: application to recombinant yeast co-consuming glucose and xylose. Biotechnol Bioeng 103: 984–1002. 19449391 10.1002/bit.22332

[9] Song H-S, Ramkrishna D, Pinchuk GE, Beliaev AS, Konopka AE, et al (2012) Dynamic modeling of aerobic growth of Shewanella oneidensis. Predicting triauxic growth, flux distributions, and energy requirement for growth. Metab Eng 15: 25–33. 23022551 10.1016/j.ymben.2012.08.004

[10] Edwards JS, Ibarra RU, Palsson BO (2001) In silico predictions of Escherichia coli metabolic capabilities are consistent with experimental data. Nat Biotechnol 19: 125–130. 11175725 10.1038/84379

[11] Zamorano F, Van de Wouwer A, Jungers RM, Bastin G (2013) Dynamic metabolic models of CHO cell cultures through minimal sets of elementary flux modes. J Biotechnol 164: 409–422. 22698821 10.1016/j.jbiotec.2012.05.005

[12] Song H-S, Ramkrishna D (2009) When is the Quasi-Steady-State Approximation Admissible in Metabolic Modeling? When Admissible, What Models are Desirable? Ind Eng Chem Res 48: 7976–7985.

[13] Wijffels RH, Barbosa MJ (2010) An Outlook on Microalgal Biofuels. Science (80-) 329: 796–799. 10.1126/science.1189003 20705853

[14] Lardon L, Helias A, Sialve B, Steyer J, Bernard O (2009) Life-cycle assessment of biodiesel production from microalgae. Environ Sci Technol 43: 6475–6481. 19764204 10.1021/es900705j

[15] Bernard O (2011) Hurdles and challenges for modelling and control of microalgae for CO2 mitigation and biofuel production. J Process Control 21: 1378–1389.

[16] Lacour T, Sciandra A, Talec A, Mayzaud P, Bernard O (2012) Diel Variations of Carbohydrates and Neutral Lipids in Nitrogen-Sufficient and Nitrogen-Starved Cyclostat Cultures of Isochrysis Sp. J Phycol 48: 966–975. 27009006 10.1111/j.1529-8817.2012.01177.x

[17] Knoop H, Gründel M, Zilliges Y, Lehmann R, Hoffmann S, et al (2013) Flux Balance Analysis of Cyanobacterial Metabolism: The Metabolic Network of Synechocystis sp. PCC 6803. PLoS Comput Biol 9: 1–15. 10.1371/journal.pcbi.1003081 PMC3699288 23843751

[18] Klamt S, Stelling J (2003) Two approaches for metabolic pathway analysis? Trends Biotechnol 21: 64–69. 12573854 10.1016/s0167-7799(02)00034-3

[19] Young JD, Ramkrishna D (2007) On the matching and proportional laws of cybernetic models. Biotechnol Prog 23: 83–99. 17269675 10.1021/bp060176q

[20] Bendif EM, Probert I, Schroeder DC, Vargas C de (2013) On the description of Tisochrysis lutea gen. nov. sp. nov. and Isochrysis nuda sp. nov. in the Isochrysidales, and the transfer of Dicrateria to the Prymnesiales (Haptophyta). J Appl Phycol 25: 1763–1776.

[21] Yang C, Hua Q, Shimizu K (2000) Energetics and carbon metabolism during growth of microalgal cells under photoautotrophic, mixotrophic and cyclic light-autotrophic/dark-heterotrophic conditions. Biochem Eng J 6: 87–102. 10959082 10.1016/s1369-703x(00)00080-2

[22] Boyle NR, Morgan JA (2009) Flux balance analysis of primary metabolism in Chlamydomonas reinhardtii. BMC Syst Biol 3: 1–14. 19128495 10.1186/1752-0509-3-4 PMC2628641

[23] Manichaikul A, Ghamsari L, Hom E, Chin C, Murray R, et al (2009) Metabolic network analysis integrated with transcript verification for sequenced genomes. Nat Methods 6: 589–592. 19597503 10.1038/nmeth.1348 PMC3139173

[24] Kliphuis A, Klok AJ, Martens DE, Lamers PP, Janssen M, et al (2012) Metabolic modeling of Chlamydomonas reinhardtii: energy requirements for photoautotrophic growth and maintenance. J Appl Phycol 24: 253–266. 22427720 10.1007/s10811-011-9674-3 PMC3289792

[25] Chang RL, Ghamsari L, Manichaikul A, Hom EFY, Balaji S, et al (2011) Metabolic network reconstruction of Chlamydomonas offers insight into light-driven algal metabolism. Mol Syst Biol 7: 1–13. 10.1038/msb.2011.52 PMC3202792 21811229

[26] Cogne G, Rügen M, Bockmayr A, Titica M, Dussap C-G, et al (2011) A model-based method for investigating bioenergetic processes in autotrophically growing eukaryotic microalgae: application to the green algae Chlamydomonas reinhardtii. Biotechnol Prog 27: 631–640. 21567987 10.1002/btpr.596

[27] Dal'Molin CGDO, Quek L-E, Palfreyman RW, Nielsen LK (2011) AlgaGEM-a genome-scale metabolic reconstruction of algae based on the Chlamydomonas reinhardtii genome. BMC Genomics 12 Suppl 4: 1–10. 10.1186/1471-2164-12-S4-S5 PMC3287588 22369158

[28] Krumholz EW, Yang H, Weisenhorn P, Henry CS, Libourel IGL (2012) Genome-wide metabolic network reconstruction of the picoalga Ostreococcus. J Exp Bot 63: 2353–2362. 22207618 10.1093/jxb/err407

[29] Terzer M, Stelling J (2008) Large-scale computation of elementary flux modes with bit pattern trees. Bioinformatics 24: 2229–2235. 18676417 10.1093/bioinformatics/btn401

[30] Williams PJLB, Laurens LML (2010) Microalgae as biodiesel & biomass feedstocks: Review & analysis of the biochemistry, energetics & economics. Energy Environ Sci: 554–590.

[31] Perry JJ, Staley JT, Lory S (2004) Biosynthèse des monomères. Microbiologie, cours et questions de révision. Paris: Dunod. pp. 206–228.

[32] Chisti Y (2007) Biodiesel from microalgae. Biotechnol Adv 25: 294–306. 17350212 10.1016/j.biotechadv.2007.02.001

[33] Liu B, Benning C (2012) Lipid metabolism in microalgae distinguishes itself. Curr Opin Biotechnol 24: 300–309. 22981869 10.1016/j.copbio.2012.08.008

[34] Guest JS, van Loosdrecht MCM, Skerlos SJ, Love NG (2013) Lumped Pathway Metabolic Model of Organic Carbon Accumulation and Mobilization by the Alga Chlamydomonas reinhardtii. Environ Sci Technol 47: 3258–3267. 23452258 10.1021/es304980y

[35] Zamorano F, Van de Wouwer A, Bastin G (2010) A detailed metabolic flux analysis of an underdetermined network of CHO cells. J Biotechnol 150: 497–508. 20869402 10.1016/j.jbiotec.2010.09.944

[36] Cheung CYM, Williams TCR, Poolman MG, Fell DA, Ratcliffe RG, et al (2013) A method for accounting for maintenance costs in flux balance analysis improves the prediction of plant cell metabolic phenotypes under stress conditions. Plant J 75: 1050–1061. 23738527 10.1111/tpj.12252

[37] Nelder J, Mead R (1965) A simplex method for function minimization. Comput J 7: 308–313.

[38] Ross O, Geider R (2009) New cell-based model of photosynthesis and photo-acclimation: accumulation and mobilisation of energy reserves in phytoplankton. Mar Ecol Prog Ser 383: 53–71.

[39] Mocquet C, Sciandra A, Talec A, Bernard O (2013) Cell cycle implication on nitrogen acquisition and synchronization in Thalassiosira weissflogii (Bacillariophyceae). J Phycol 49: 371–380. 27008523 10.1111/jpy.12045

[40] Willey J, Sherwood L, Woolverton C (2008) Metabolism: Energy, Enzymes, and Regulation. Prescott, Harley and Klein's Microbiology. Mc Graw Hill higher Education. pp. 167–190.

[41] Ovádi J, Saks V (2004) On the origin of intracellular compartmentation and organized metabolic systems. Mol Cell Biochem 256–257: 5–12. 10.1023/b:mcbi.0000009855.14648.2c 14977166

[42] Schuster S, Pfeiffer T, Moldenhauer F, Koch I, Dandekar T (2002) Exploring the pathway structure of metabolism: decomposition into subnetworks and application to Mycoplasma pneumoniae. Bioinformatics 18: 351–361. 11847093 10.1093/bioinformatics/18.2.351

[43] Larhlimi A, David L, Selbig J, Bockmayr A (2012) F2C2: a fast tool for the computation of flux coupling in genome-scale metabolic networks. BMC Bioinformatics 13: 1–9. 22524245 10.1186/1471-2105-13-57 PMC3515416

[44] Verwoerd WS (2011) A new computational method to split large biochemical networks into coherent subnets. BMC Syst Biol 5: 1–25. 21294924 10.1186/1752-0509-5-25 PMC3045323

[45] Barabási A-L, Oltvai ZN (2004) Network biology: understanding the cell's functional organization. Nat Rev Genet 5: 101–113. 14735121 10.1038/nrg1272

[46] Akaike H (1974) A new look at the statistical model identification. IEEE Trans Automat Contr 19: 716–723.

[47] Song H-S, Ramkrishna D (2009) Reduction of a set of elementary modes using yield analysis. Biotechnol Bioeng 102: 554–568. 18853408 10.1002/bit.22062

[48] Song H-S, Ramkrishna D (2010) Prediction of metabolic function from limited data: Lumped hybrid cybernetic modeling (L-HCM). Biotechnol Bioeng 106: 271–284. 20148411 10.1002/bit.22692

[49] Ramkrishna D, Song H (2012) Dynamic models of metabolism: Review of the cybernetic approach. AIChE J 58: 986–997.

[50] Curien G, Bastien O, Robert-Genthon M, Cornish-Bowden A, Cárdenas ML, et al (2009) Understanding the regulation of aspartate metabolism using a model based on measured kinetic parameters. Mol Syst Biol 5: 271. 19455135 10.1038/msb.2009.29 PMC2694679

[51] Zomorrodi AR, Maranas CD (2012) OptCom: a multi-level optimization framework for the metabolic modeling and analysis of microbial communities. PLoS Comput Biol 8: 1–13. 22319433 10.1371/journal.pcbi.1002363 PMC3271020

[52] Rügen M, Bockmayr A, Legrand J, Cogne G (2012) Network reduction in metabolic pathway analysis: Elucidation of the key pathways involved in the photoautotrophic growth of the green alga Chlamydomonas reinhardtii. Metab Eng 14: 458–467. 22342232 10.1016/j.ymben.2012.01.009

[53] Packer A, Li Y, Andersen T, Hu Q, Kuang Y, et al (2011) Growth and neutral lipid synthesis in green microalgae: a mathematical model. Bioresour Technol 102: 111–117. 20619638 10.1016/j.biortech.2010.06.029

[54] Mairet F, Bernard O, Lacour T, Sciandra A (2011) Modelling microalgae growth in nitrogen limited photobiorector for estimating biomass, carbohydrate and neutral lipid productivities. Proc 18th IFAC World Congr 1: 1–6.

[55] Quinn J, de Winter L, Bradley T (2011) Microalgae bulk growth model with application to industrial scale systems. Bioresour Technol 102: 5083–5092. 21324679 10.1016/j.biortech.2011.01.019

[56] Tevatia R, Demirel Y, Blum P (2012) Kinetic Modeling of Photoautotropic Growth and Neutral Lipid Accumulation in terms of Ammonium Concentration in Chlamydomonas reinhardtii. Bioresour Technol 119: 419–424. 22727606 10.1016/j.biortech.2012.05.124

[57] Yang J, Rasa E, Tantayotai P, Scow KM, Yuan H, et al (2011) Mathematical model of Chlorella minutissima UTEX2341 growth and lipid production under photoheterotrophic fermentation conditions. Bioresour Technol 102: 3077–3082. 21115343 10.1016/j.biortech.2010.10.049 PMC3267903

[58] Fleck-Schneider P, Lehr F, Posten C (2007) Modelling of growth and product formation of Porphyridium purpureum. J Biotechnol 132: 134–141. 17658642 10.1016/j.jbiotec.2007.05.030

[59] Mairet F, Bernard O, Masci P, Lacour T, Sciandra A (2011) Modelling neutral lipid production by the microalga Isochrysis aff. galbana under nitrogen limitation. Bioresour Technol 102: 142–149. 20656476 10.1016/j.biortech.2010.06.138

[60] Bastin G, Dochain D (1990) On-line estimation and adaptive control of bioreactors. Amsterdam: Elseviers.

[61] Bernard O, Bastin G (2005) Identification of reaction networks for bioprocesses: determination of a partially unknown pseudo-stoichiometric matrix. Bioprocess Biosyst Eng 27: 293–301. 15995850 10.1007/s00449-005-0407-3

[62] Bernard O, Bastin G (2005) On the estimation of the pseudo-stoichiometric matrix for macroscopic mass balance modelling of biotechnological processes. Math Biosci 193: 51–77. 15681276 10.1016/j.mbs.2004.10.004

[63] Koutinas M, Kiparissides A, Silva-Rocha R, Lam M-C, Martins Dos Santos V a P, et al (2011) Linking genes to microbial growth kinetics: an integrated biochemical systems engineering approach. Metab Eng 13: 401–413. 21315172 10.1016/j.ymben.2011.02.001

[64] Segre D, Vitkup D, Church G (2002) Analysis of optimality in natural and perturbed metabolic networks. Proc Natl Acad Sci 99: 15112–15117. 12415116 10.1073/pnas.232349399 PMC137552

[65] Burgard AP, Pharkya P, Maranas CD (2003) Optknock: a bilevel programming framework for identifying gene knockout strategies for microbial strain optimization. Biotechnol Bioeng 84: 647–657. 14595777 10.1002/bit.10803

[66] Pharkya P, Burgard A, Maranas C (2004) OptStrain: a computational framework for redesign of microbial production systems. Genome Res 14: 2367–2376. 15520298 10.1101/gr.2872004 PMC525696

[67] Shlomi T, Berkman O, Ruppin E (2005) Regulatory on – off minimization of metabolic flux after genetic perturbations. PNAS 102: 7698–7700. 10.1073/pnas.0406346102 PMC1140402 15897462

[68] Kim J, Reed JL (2010) OptORF: Optimal metabolic and regulatory perturbations for metabolic engineering of microbial strains. BMC Syst Biol 4: 1–19. 20426856 10.1186/1752-0509-4-53 PMC2887412

[69] Zanghellini J, Ruckerbauer DE, Hanscho M, Jungreuthmayer C (2013) Elementary flux modes in a nutshell: properties, calculation and applications. Biotechnol J 8: 1009–1016. 23788432 10.1002/biot.201200269

