# Supplementary figures and images for: DRUM: A New Framework for Metabolic Modeling under Non-Balanced Growth. Application to the Carbon Metabolism of Unicellular Microalgae
Source: PLoS One. 2014 Aug 8;9(8):e104499. doi: 10.1371/journal.pone.0104499 (PMC4126706; doi:10.1371/journal.pone.0104499)

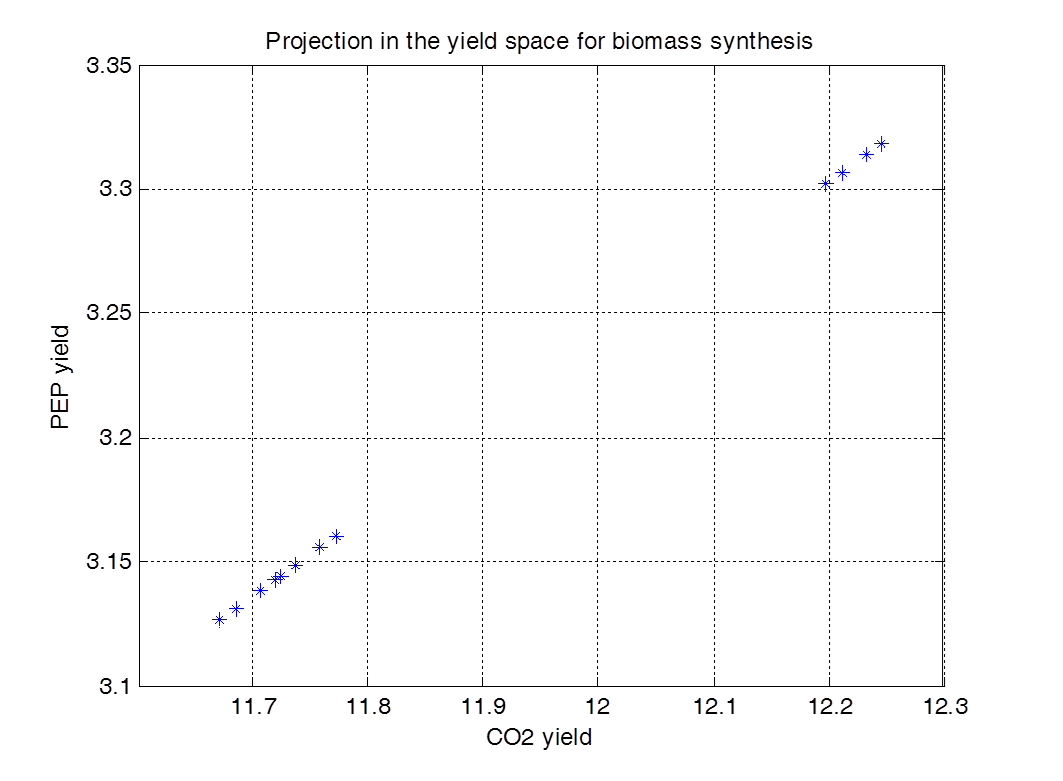

Supplement: Figure S1 — Projection of elementary flux modes obtained from the biomass synthesis sub-network in the PEP/CO2 yield space. The reduction of the biomass synthesis sub-network leads to 30 macroscopic reactions, in which 24 yields biomass. In terms of carbon, the 24 macroscopic reactions were only different in their consumption of PEP and hence their production of CO2. A projection in the yield space PEP = f(CO2) reveals two distinct metabolic behaviors. (TIF) [file pone.0104499.s001.tif]

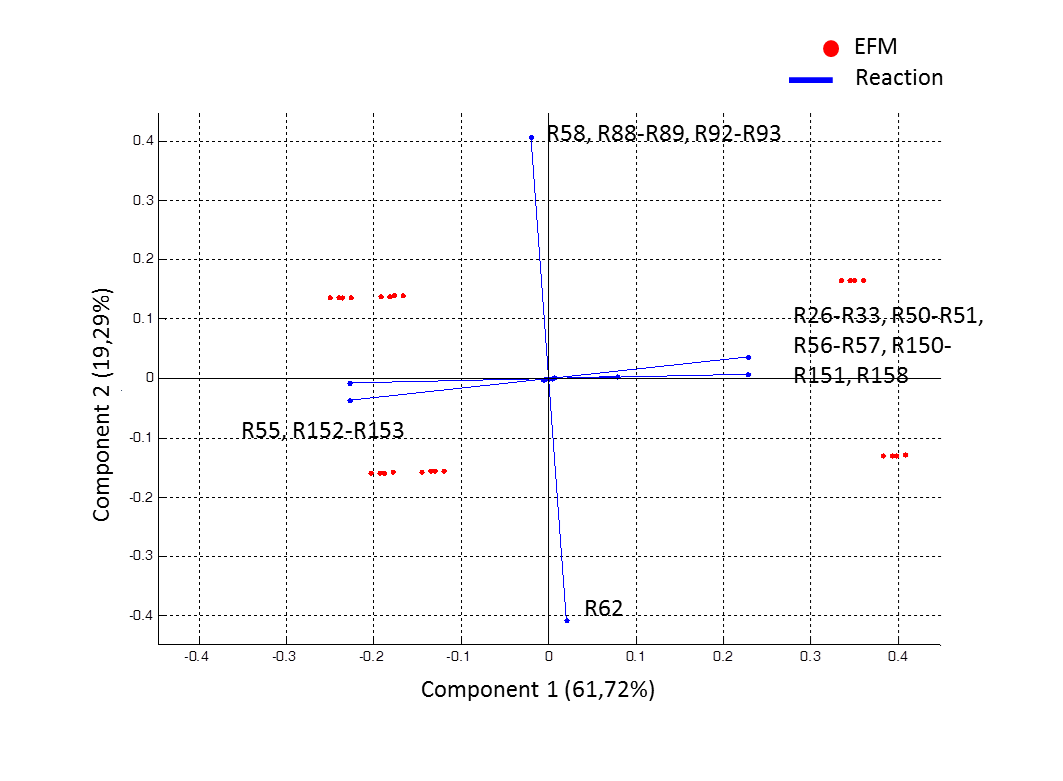

Supplement: Figure S2 — Principal component analysis of the elementary flux modes obtained from the biomass synthesis sub-network. The difference in the PEP/CO2 yield is mainly due to two metabolic functions (incorporation of nitrogen (x-axis) and alanine synthesis (y-axis)) that can be performed thanks to different pathways, some less energy-efficient than others explaining the difference in CO2 production. (TIF) [file pone.0104499.s002.tif]

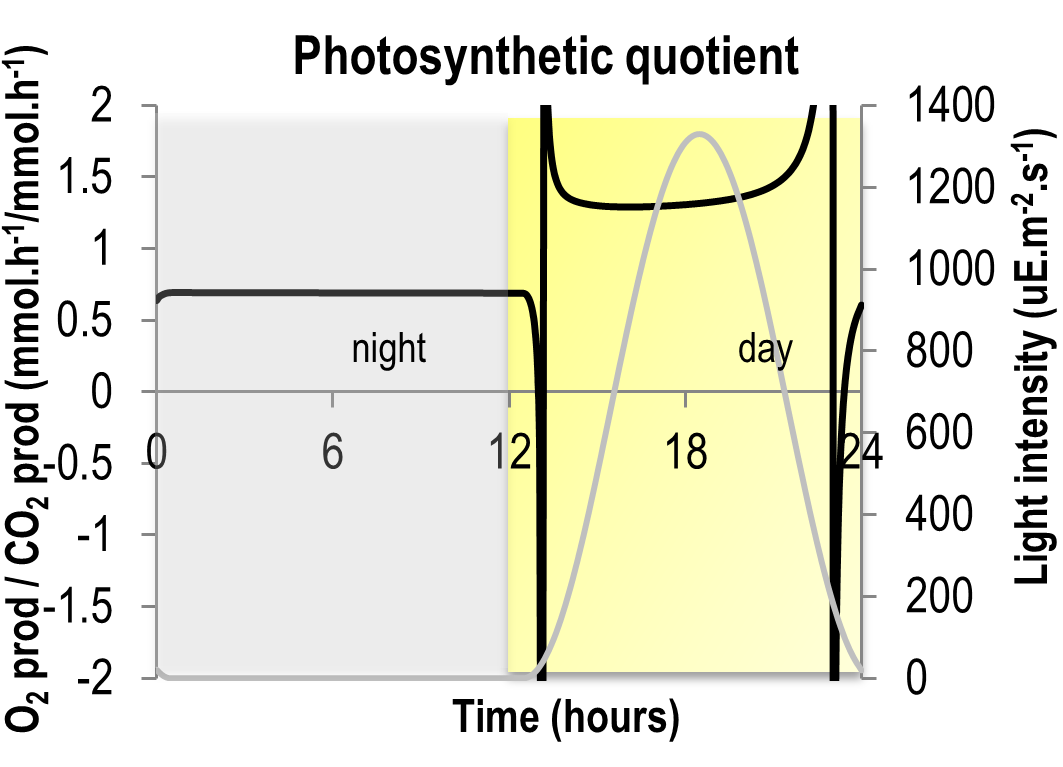

Supplement: Figure S3 — Predicted photosynthetic quotient during a day/night cycle. The quotient varies between 1.29 and 1.60, depending on the light intensity, which agrees with the typical range of 1.0–1.8 for algae [22]. (TIF) [file pone.0104499.s003.tif]

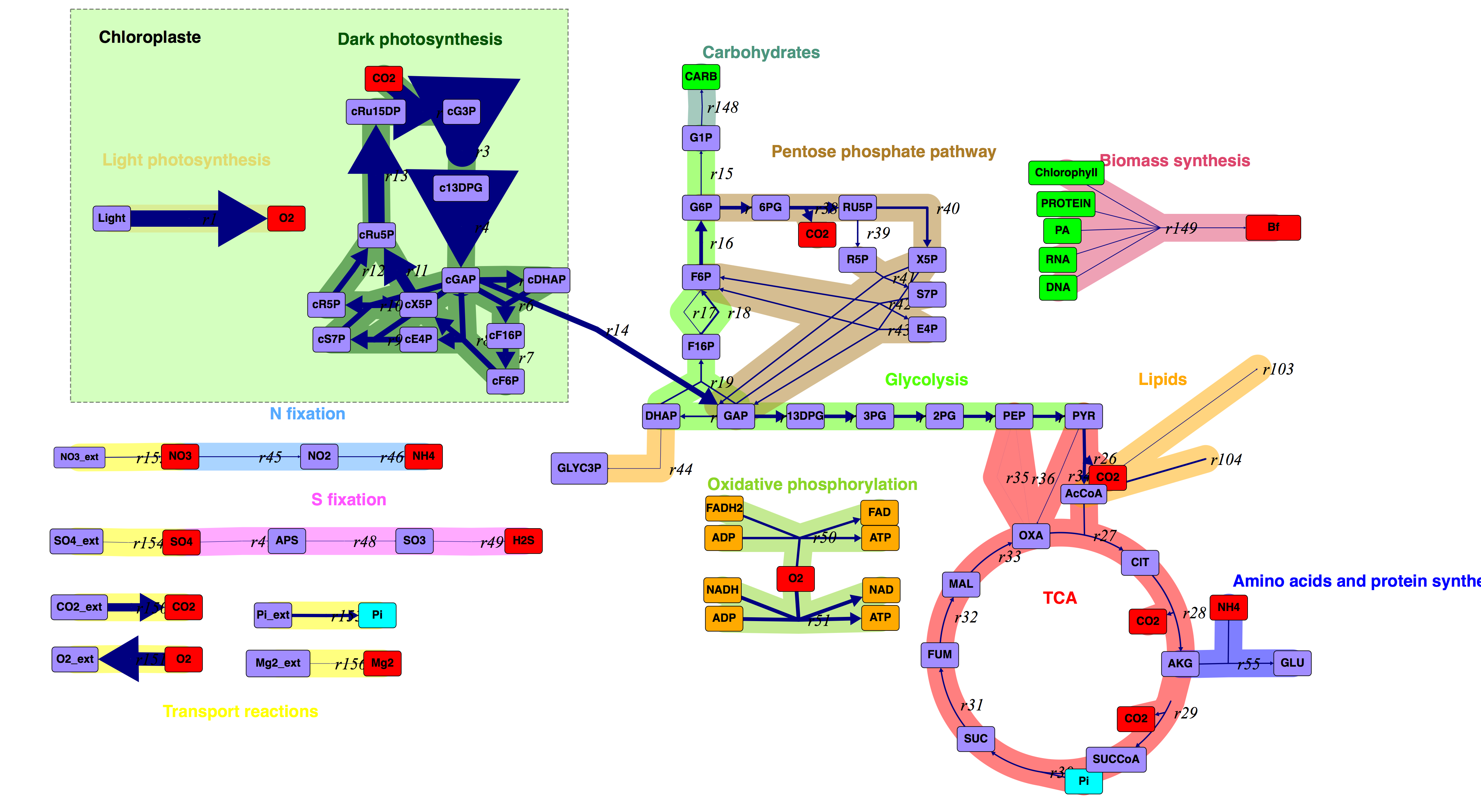

Supplement: Figure S4 — Metabolic fluxes of the core network at midday (18 h). (PNG) [file pone.0104499.s004.png]

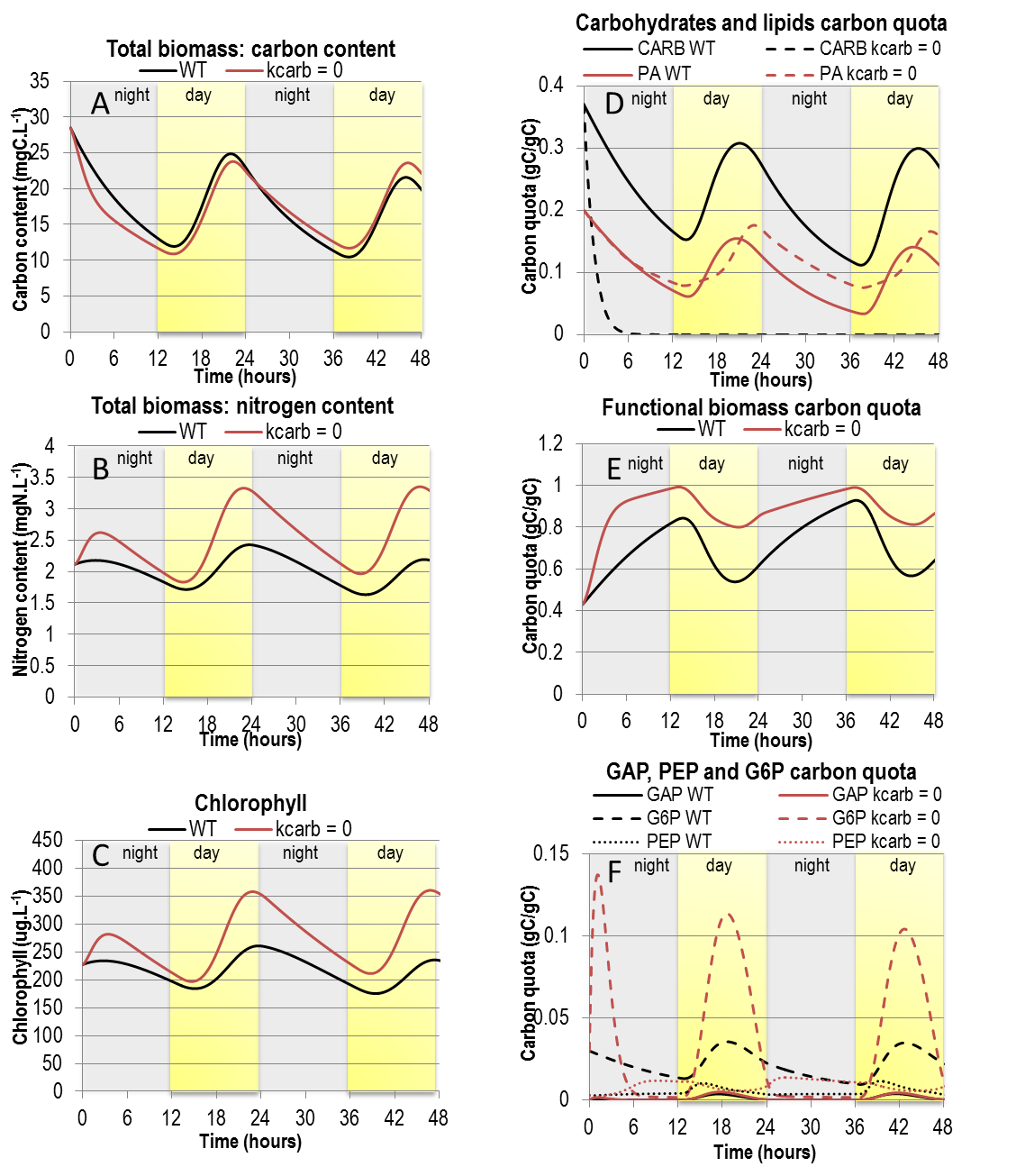

Supplement: Figure S5 — Comparison of the wild type and MR6-deficient in silico models. The two models were then simulated for 48 h, one with kcarb = 0 h−1.mM B−1, the other one with kcarb = 70.00 h−1.mM B−1. The dilution rate and the incoming substrate concentrations were set at 1 days−1 and 4.018 mgN.L−1. (PNG) [file pone.0104499.s005.png]
