# Supplementary material for: DRUM: A New Framework for Metabolic Modeling under Non-Balanced Growth. Application to the Carbon Metabolism of Unicellular Microalgae
Source: PLoS One. 2014 Aug 8;9(8):e104499. doi: 10.1371/journal.pone.0104499 (PMC4126706; doi:10.1371/journal.pone.0104499)
Supplement: File S1 — Detailed metabolic network reconstruction process of Tisochrysis lutea ; list of reactions and metabolites; analysis of the whole metabolic network; list of sub-networks; list of macroscopic reactions obtained for the biomass synthesis sub-network. (PDF) [file pone.0104499.s006.pdf]

File S1

1 Metabolic network construction of *Tisochrysis lutea*

The metabolic network of Kliphuis et al. in [1] was used as a starting point since this metabolic network is rather small and generic (152 metabolites, 160 reactions) and represents only the core metabolic network common to eukaryote microalgae (photosynthesis, glycolysis, pentose phosphate pathway, TCA cycle, oxidative phosphorylation, carbohydrate, lipids, protein, DNA, RNA, chlorophyll and biomass synthesis). Indeed, their representation is in large agreement with others metabolic network reconstruction of eukaryotic microalgae [1–8]. Some minor modifications of the metabolic network were performed (e.g.: transport reaction from the chloroplast) and reactions of macromolecules synthesis (lipids, proteins, DNA, RNA and biomass) were determined using experimental data of [9].

1.1 Lipids synthesis reaction

Lipids are classically represented as phosphatic acids (PA), composed of a glycerol 3-phosphate molecule with two average acetyl-ACP tails. The average acetyl-ACP chain was determined thanks to the molar fractions of the various fatty acids of *Isochrysis galbana* from [10]. This yielded the metabolic equations:

```
ACP + AcCoA + H <--> AcACP + CoA
ATP + AcCoA + CO2 + H2O <--> ADP + H + MalCoA + Pi
ACP + MalCoA <--> CoA + MalACP
12 H + 12 NADPH + 6 MalACP + AcACP <--> C14:0ACP + 6 ACP + 6 CO2 + 6 H2O + 12 NADP
C14:0ACP + H + NADH + O2 <--> C14:1ACP + NAD + 2 H2O
14 H + 14 NADPH + 7 MalACP + AcACP <--> C16:0ACP + 7 ACP + 7 CO2 + 7 H2O + 14 NADP
C16:0ACP + H + NADH + O2 <--> C16:1ACP + NAD + 2 H2O
C16:1ACP + H + NADH + O2 <--> C16:2ACP + NAD + 2 H2O
C16:2ACP + H + NADH + O2 <--> C16:3ACP + NAD + 2 H2O
16 H + 16 NADPH + 8 MalACP + AcACP <--> C18:0ACP + 8 ACP + 8 CO2 + 8 H2O + 16 NADP
C18:0ACP + H + NADH + O2 <--> C18:1ACP + NAD + 2 H2O
C18:1ACP + H + NADH + O2 <--> C18:2ACP + NAD + 2 H2O
C18:2ACP + H + NADH + O2 <--> C18:3ACP + NAD + 2 H2O
C18:3ACP + H + NADH + O2 <--> C18:4ACP + NAD + 2 H2O
18 H + 18 NADPH + 9 MalACP + AcACP <--> C20:0ACP + 9 ACP + 9 CO2 + 9 H2O + 18 NADP
C20:0ACP + H + NADH + O2 <--> C20:1ACP + NAD + 2 H2O
C20:1ACP + H + NADH + O2 <--> C20:2ACP + NAD + 2 H2O
C20:2ACP + H + NADH + O2 <--> C20:3ACP + NAD + 2 H2O
C20:3ACP + H + NADH + O2 <--> C20:4ACP + NAD + 2 H2O
C20:4ACP + H + NADH + O2 <--> C20:5ACP + NAD + 2 H2O
GLYC3P + 0.524 C14:0ACP + 0.020 C14:1ACP + 0.222 C16:0ACP + 0.096 C16:1ACP + 0.016 C16:3ACP + 0.012
C18:0ACP + 0.724 C18:1ACP + 0.106 C18:2ACP + 0.102 C18:3ACP + 0.142 C18:4ACP + 0.036 C20:5ACP <--> PA +
2 ACP + 2 H
```

1.2 Protein synthesis reaction

Proteins are represented as an average protein determined thanks to the molar fraction of each amino acids present in the cell. Brown in [11] showed that the average composition of amino acids in microalgae does not significantly vary between microalgae species. Hence, we assume that the average composition of amino acids in *Tisochrysis lutea* is similar to the one of *Chlamydomonas reinhardtii*, and we used the protein synthesis reaction of [1]. In a similar way, we assumed that DNA and RNA were not significantly varying between eukaryote microalgae species and hence we also took the DNA and RNA synthesis reactions from [1].

1.3 Biomass synthesis equation

As we take into account accumulation of intracellular metabolites in our methodology, biomass B has no longer the same significance. Indeed, the biomass is usually represented as an average composition of macromolecules present in the cell. For example, in the case of *Chlamydomonas reinhardtii*, the biomass is in average composed of 64.17% of protein, 27.13% of carbohydrates, 4.53% of lipids, 3.05% of RNA, 1.02% of Chlorophyll and 0.11% DNA (molar ratios). But this average representation of the biomass constrains carbohydrates and lipids to a fixed percentage, which is not the case for microalgae under day/night cycles [9]. Hence lipids and carbohydrates are no longer part of the biomass equation and instead are metabolites authorized to accumulate (A). Biomass is then uniquely composed of proteins, DNA, RNA and Chlorophyll, which we rename as functional biomass. Biomass composed of all the macromolecules of the cell is then the sum of metabolites authorized to accumulate A and functional biomass B:

$$X_Z(t) = \sum_A Z_A * A(t) + Z_B * B(t)$$

where Z correspond to a chemical element ($Z \in \{C; N; O; H; P; S; \dots\}$), Z_A and Z_B corresponds to the number of chemical element Z per mole of accumulating metabolites A and biomass B, $A(t)$ and $B(t)$ correspond to the concentrations of A and B at time t, and $X_Z(t)$ correspond to the concentration of chemical element Z in total biomass X at time t.

DNA and RNA contents were not measured in [9]. However, Geider and Laroche in [12] have shown that DNA and RNA contents do not vary much between microalgae species under nutrient-replete conditions. Hence, we assume that the DNA and RNA contents are similar between *Chlamydomonas reinhardtii* and *Tisochrysis lutea*.

Chlorophyll content was measured, yielding a mean value of $0.0077\ gN.gN^{-1}$, which is significantly lower than the value reported in [1] ($0.0410\ gN.gN^{-1}$). This difference can be easily explained by the difference of light intensity applied in the two experiments: up to 1500 $\mu E.m^{-2}.s^{-1}$ in [9] against 100 $\mu E.m^{-2}.s^{-1}$ in [1]. Because of photoadaptation, the content of chlorophyll per unit of biomass decreases when light intensity increases [13].

Functional biomass can be indirectly deduced from the experimental data, thanks to a mass-balance on intracellular carbon and nitrogen:

$X_C = C_A * A + C_B * B \Rightarrow B = \frac{(X_C - C_A * A)}{C_B} \approx \frac{X_C - C_{CARB} * CARB - C_{PA} * PA}{C_B}$, because GAP, PEP and G6P contributions in terms of carbon are assumed negligible.

$X_N = N_A * A + N_B * B = N_B * B$, because all nitrogen is in the form of functional biomass.

- with X_C total intracellular carbon ($mM\ C.L^{-1}$)
- C_A: number of carbon atoms in a molecule of A
- A: concentration of A ($mM.L^{-1}$)
- C_B: number of carbon atoms in a molecule of B
- B: concentration of B ($mM.L^{-1}$)
- C_{CARB}: number of carbon atoms in a molecule of $CARB$
- $CARB$: concentration of $CARB$ ($mM.L^{-1}$)
- C_{PA}: number of carbon atoms in a molecule of PA
- PA: concentration of PA ($mM.L^{-1}$)
- X_N: total intracellular nitrogen
- N_A: number of nitrogen atoms in a molecule of A
- N_B: number of nitrogen atoms in a molecule of B

Finally, protein content was assumed to be the same content as in [1]. Hence, we obtained the following biomass equation:

32.687 ATP + 32.687 H2O + 0.9504 PROTEIN + 0.0028 Chlorophyll + 0.0452 RNA + 0.0016 DNA --> B + 32.687 H + 32.687 ADP + 32.687 Pi

The C/N ratio for the functional biomass obtained is 3.11. This value is lower than the average experimental value (5.81). However, during the experiment only triacylglycerol (TAGs) lipids were

measured. To take into account others lipids present in the cell (e.g., cell walls) and hence match the experimental C/N ratio, a quantity of PA was added in the functional biomass synthesis equation:

32.687 ATP + 32.687 H2O + 0.8456 PROTEIN + 0.0026 Chlorophyll + 0.0402 RNA + 0.0014 DNA + 0.1103 PA --> B + 32.687 H + 32.687 ADP + 32.687 Pi

1.4 Other modifications

We removed glycerol synthesis and excretion reactions, because *Tisochrysis lutea* produces very low quantities of glycerol. Starch synthesis was lumped in the overall carbohydrates pathway. The reaction of conversion of NADPH to NADH was also removed, to prevent the non-use of the pentose phosphate pathway, which is often a problem encountered in FBA [14]. We dropped as well photorespiration which was experimentally found negligible [15], and changed the quantum yield of photosynthesis to 10 photons per CO_2 incorporated instead of 8, because not all light is taken up by the photosystems and 10 is the predominant value that can be found in literature [1].

In addition, the irreversible reaction R104, which converts Acetyl-Coenzyme A to Malonyl-Coenzyme, was found to prevent consumption of PAs during the night. Thus, R104 was assumed reversible. Finally, GAP was assumed exported from the chloroplast, instead of DHAP, in accordance with [3].

1.5 Chemical Element Composition of the Macromolecules

Below is presented the chemical element composition of accumulating metabolites *A* and the macromolecules of the cell of the metabolic network to *Tisochyris Lutea.*

	C	H	O	N	P	S
**GAP**	3	7	6	0	1	0
**G6P**	6	13	9	0	1	0
**PEP**	3	5	6	0	1	0
**CARB**	6	10	5	0	0	0
**PA**	36.2	63.4	8	0	1	0
**PROTEIN**	4.8	7.6	1.5	1.3	0	0.03
**DNA**	9.7	12.2	7	3.8	1	0
**RNA**	9.5	12.8	8	3.8	1	0
**Chlorophylle**	55	72	5	4	0	0
**B**	8.59	14.13	2.49	1.26	0.15	0.025

2 List of reactions

Light step of photosynthesis

R1 10 Light + 3 ADP + 3 Pi + H + 2 cNADP --> O2 + H2O + 2 cNADPH + 3 ATP

Dark step of photosynthesis

R2 CO2 + H2O + cRu15DP --> 2 cG3P

R3 ATP + cG3P --> ADP + H + c13DPG

R4 H + cNADPH + c13DPG <--> cNADP + Pi + cGAP

R5 cGAP <--> cDHAP

R6 cDHAP + cGAP <--> cF16P

R7 H2O + cF16P <--> Pi + cF6P

R8 cF6P + cGAP <--> cE4P + cX5P

R9 H2O + cE4P + cGAP <--> Pi + cS7P

R10 cGAP + cS7P <--> cR5P + cX5P

R11 cX5P <--> cRu5P

R12 cR5P <--> cRu5P

R13 ATP + cRu5P --> ADP + H + cRu15DP

R14 cGAP <--> GAP

Glycolysis

R15 G6P <--> G1P

R16 F6P <--> G6P

R17 ATP + F6P --> ADP + F16P + H

R18 F16P + H2O --> F6P + Pi

R19 DHAP + GAP <--> F16P

R20 DHAP <--> GAP

R21 GAP + NAD + Pi <--> 13DPG + H + NADH

R22 13DPG + ADP <--> 3PG + ATP

R23 3PG <--> 2PG

R24 2PG <--> H2O + PEP

R25 ADP + H + PEP --> ATP + PYR

Tricarboxylic acid cycle

R26 CoA + NAD + PYR --> AcCoA + CO2 + NADH

R27 AcCoA + H2O + OXA <--> CIT + CoA + H

R28 CIT + NAD <--> AKG + CO2 + NADH

R29 AKG + CoA + NAD --> CO2 + NADH + SUCCoA

R30 ADP + Pi + SUCCoA <--> ATP + CoA + SUC

R31 FAD + SUC <--> FADH2 + FUM

R32 FUM + H2O <--> MAL

R33 FAD + MAL <--> FADH2 + OXA

R34 ATP + CO2 + H2O + PYR --> ADP + OXA + Pi + 2 H

R35 ATP + OXA --> ADP + CO2 + PEP

R36 CO2 + H2O + PEP <--> H + OXA + Pi

Pentose phosphate pathway

R37 G6P + H2O + NADP <--> 6PG + NADPH + 2 H

R38 6PG + NADP <--> CO2 + NADPH + RU5P

R39 RU5P <--> R5P

R40 RU5P <--> X5P

R41 R5P + X5P <--> GAP + S7P

R42 GAP + S7P <--> E4P + F6P

R43 F6P + GAP <--> E4P + X5P

Glycerol synthesis

R44 GLYC3P + NAD <--> DHAP + H + NADH

N fixation

R45 H + NADH + NO3 <--> H2O + NAD + NO2

R46 5 H + 3 NADPH + NO2 <--> NH4 + 2 H2O + 3 NADP

S fixation

R47 ATP + SO4 --> APS + PPi

R48 APS + NADH --> AMP + NAD + SO3

R49 5 H + 3 NADPH + SO3 <--> H2S + 3 H2O + 3 NADP

Oxidative phosphorylation

R50 1.5 ADP + 1.5 H + 1.5 Pi + FADH2 + 0.5 O2 --> FAD + 1.5 ATP + 2.5 H2O

R51 3.5 H + 2.5 ADP + 2.5 Pi + NADH + 0.5 O2 --> NAD + 2.5 ATP + 3.5 H2O

R52 H2O + PPi --> H + 2 Pi

R53 AMP + ATP --> 2 ADP

R54 ATP + H2O --> ADP + H + Pi + MAINT

Amino acids and protein synthesis

R55 AKG + H + NADPH + NH4 --> GLU + H2O + NADP

R56 ATP + GLU + NH4 --> ADP + GLN + H + Pi

R57 AKG + GLN + H + NADPH <--> NADP + 2 GLU

R58 3PG + GLU + H2O + NAD <--> AKG + H + NADH + Pi + SER

R59 SER --> NH4 + PYR

R60 AcCoA + H2S + SER <--> Ace + CYS + CoA + H

R61 ATP + Ace + CoA --> ADP + AcCoA + Pi

R62 GLU + PYR --> AKG + ALA

R63 H + THR <--> 2-oxobutan + NH4

R64 2-oxobutan + GLU + H + NADPH + PYR <--> AKG + CO2 + H2O + ILE + NADP

R65 2 H + ALA + NADPH + PYR <--> CO2 + H2O + NADP + VAL

R66 2 PYR + AcCoA + GLU + H + NAD + NADPH <--> AKG + CoA + LEU + NADH + NADP + 2 CO2

R67 2 PEP + ATP + E4P + NADPH --> ADP + CHO + NADP + 4 Pi

R68 CHO <--> PRE

R69 GLU + H + PRE <--> AKG + CO2 + H2O + PHE

R70 GLU + NAD + PRE <--> AKG + CO2 + NADH + TYR

R71 CHO + GLN <--> ANTH + GLU + H + PYR

R72 ANTH + H + PRPP + SER <--> CO2 + GAP + PPi + TRYP + 2 H2O

R73 3 H2O + 2 NAD + ATP + GLN + PRPP --> AICAR + AKG + HIS + Pi + 2 NADH + 2 PPi + 5 H

R74 GLU + OXA <--> AKG + ASP

R75 ASP + ATP + GLN + H2O --> ADP + ASN + GLU + H + Pi

R76 2 ATP + 2 H2O + CO2 + GLN --> CaP + GLU + Pi + 2 ADP + 3 H

R77 2 GLU + ASP + ATP + CaP + NADH --> AKG + AMP + ARG + FUM + H2O + NAD + PPi + Pi

R78 3 H + 2 NADH + GLU <--> PRO + 2 H2O + 2 NAD

R79 AKG + O2 + PRO <--> CO2 + HydPro + SUC

R80 ASP + ATP + H + NADPH --> ADP + ASA + NADP + Pi

R81 2 H + ASA + GLU + NADH + PYR <--> AKG + DAP + H2O + NAD

R82 DAP <--> CO2 + H + LYS

R83 ASA + H + NADPH <--> HSER + NADP

R84	ATP + H2O + HSER --> ADP + H + Pi + THR
R85	AcCoA + CYS + H2O + HSER <--> Ace + CoA + HCYS + H + NH4 + PYR
R86	HCYS + MTHF <--> H + MET + THF
R87	4.306 ATP + 3.306 H2O + 0.111 ALA + 0.092 GLY + 0.09 LEU + 0.061 VAL + 0.06 LYS + 0.056 PRO + 0.056 THR + 0.054 SER + 0.052 ARG + 0.052 GLN + 0.052 GLU + 0.047 ASN + 0.047 ASP + 0.041 PHE + 0.037 ILE + 0.03 TYR + 0.024 MET + 0.017 HIS + 0.012 CYS + 0.0090 HydPro + 0.0010 TRYP --> PROTEIN + 4.306 ADP + 4.306 Pi + 4.319 H
R88	GLY + H + PYR <--> ALA + glyoxylate
R89	SER + glyoxylate <--> GLY + HydPyr
R90	GLY + H2O + METHF <--> SER + THF
R91	GLY + NAD + THF <--> CO2 + METHF + NADH + NH4
R92	H + HydPyr + NADH <--> Glycerate + NAD
R93	ATP + Glycerate --> ADP + 2 H + 3PG
	**THF metabolism**
R94	ATP + R5P --> AMP + H + PRPP
R95	5FTHF + H <--> H2O + MYLTHF
R96	H2O + MYLTHF <--> H + N10FTHF
R97	ATP + FORM + THF --> ADP + N10FTHF + Pi
R98	MYLTHF + NADPH <--> METHF + NADP
R99	H + METHF + NADPH <--> MTHF + NADP
R100	5FTHF + ATP + H2O --> ADP + H + N10FTHF + Pi
R101	FORM + H + THF <--> H2O + N10FTHF
R102	DHF + H + NADPH <--> NADP + THF
	**Lipids synthesis**
R103	ACP + AcCoA + H <--> AcACP + CoA
R104	ATP + AcCoA + CO2 + H2O <--> ADP + H + MalCoA + Pi
R105	ACP + MalCoA <--> CoA + MalACP
R106	12 H + 12 NADPH + 6 MalACP + AcACP <--> C14:0ACP + 6 ACP + 6 CO2 + 6 H2O + 12 NADP
R107	C14:0ACP + H + NADH + O2 <--> C14:1ACP + NAD + 2 H2O
R108	14 H + 14 NADPH + 7 MalACP + AcACP <--> C16:0ACP + 7 ACP + 7 CO2 + 7 H2O + 14 NADP
R109	C16:0ACP + H + NADH + O2 <--> C16:1ACP + NAD + 2 H2O
R110	C16:1ACP + H + NADH + O2 <--> C16:2ACP + NAD + 2 H2O
R111	C16:2ACP + H + NADH + O2 <--> C16:3ACP + NAD + 2 H2O
R112	16 H + 16 NADPH + 8 MalACP + AcACP <--> C18:0ACP + 8 ACP + 8 CO2 + 8 H2O + 16 NADP
R113	C18:0ACP + H + NADH + O2 <--> C18:1ACP + NAD + 2 H2O
R114	C18:1ACP + H + NADH + O2 <--> C18:2ACP + NAD + 2 H2O
R115	C18:2ACP + H + NADH + O2 <--> C18:3ACP + NAD + 2 H2O
R116	C18:3ACP + H + NADH + O2 <--> C18:4ACP + NAD + 2 H2O
R117	18 H + 18 NADPH + 9 MalACP + AcACP <--> C20:0ACP + 9 ACP + 9 CO2 + 9 H2O + 18 NADP
R118	C20:0ACP + H + NADH + O2 <--> C20:1ACP + NAD + 2 H2O
R119	C20:1ACP + H + NADH + O2 <--> C20:2ACP + NAD + 2 H2O
R120	C20:2ACP + H + NADH + O2 <--> C20:3ACP + NAD + 2 H2O
R121	C20:3ACP + H + NADH + O2 <--> C20:4ACP + NAD + 2 H2O
R122	C20:4ACP + H + NADH + O2 <--> C20:5ACP + NAD + 2 H2O
R123	GLYC3P + 0.524 C14:0ACP + 0.02 C14:1ACP + 0.222 C16:0ACP + 0.096 C16:1ACP + 0.016

C16:3ACP + 0.012 C18:0ACP + 0.724 C18:1ACP + 0.106 C18:2ACP + 0.102 C18:3ACP + 0.142 C18:4ACP + 0.036 C20:5ACP <--> PA + 2 ACP + 2 H

Nucleic acids synthesis

R124 4 ATP + 2 GLN + 2 H2O + ASP + CO2 + GLY + N10FTHF + PRPP --> AICAR + FUM + PPi + THF + 2 GLU + 4 ADP + 4 Pi + 7 H

R125 ASP + CaP + H + O2 + PRPP <--> CO2 + H2O + H2O2 + PPi + Pi + UMP

R126 2 H2O2 <--> O2 + 2 H2O

R127 ATP + UMP --> ADP + UDP

R128 ATP + UDP <--> ADP + UTP

R129 ATP + GLN + H2O + UTP --> ADP + CTP + GLU + Pi + 2 H

R130 ATP + CDP <--> ADP + CTP

R131 AICAR + N10FTHF <--> H2O + IMP + THF

R132 ATP + H2O + IMP + NAD + NH4 --> AMP + GMP + NADH + PPi + 3 H

R133 ATP + GMP --> ADP + GDP

R134 ATP + GDP <--> ADP + GTP

R135 ASP + GTP + IMP <--> AMP + FUM + GDP + Pi + 2 H

R136 ATP + H + METHF + NADPH + UDP --> ADP + DHF + H2O + NADP + dTTP

R137 ATP + CDP + H + NADPH --> ADP + H2O + NADP + dCTP

R138 ATP + GDP + H + NADPH --> ADP + H2O + NADP + dGTP

R139 ATP + H + NADPH <--> H2O + NADP + dATP

R140 2.372 H2O + 1.372 ATP + 0.18 dATP + 0.18 dTTP + 0.32 dCTP + 0.32 dGTP --> DNA + PPi + 1.372 ADP + 1.372 Pi + 2.372 H

R141 1.4 H2O + 0.56 ATP + 0.34 GTP + 0.16 UTP + 0.34 CTP --> 0.4 ADP + 0.4 H + 0.4 Pi + PPi + RNA

Chlorophyll synthesis

R142 12 H + 8 ATP + 8 GLU + 8 NADPH + 2.5 O2 --> PPorphyrin + 4 NH4 + 6 CO2 + 8 AMP + 8 NADP + 8 PPi + 13 H2O

R143 18 H + 15 NADPH + 8 ATP + 4 GAP + 4 PYR --> Phytyl-PP + 4 ADP + 4 AMP + 4 CO2 + 7 PPi + 8 H2O + 15 NADP

R144 ATP + H2O + MET --> AdMET + H + PPi + Pi

R145 AdHCYS + H2O <--> Ad + HCYS

R146 ATP + Ad --> ADP + AMP + H

R147 4 NADPH + 2.5 O2 + 2 ATP + AdMET + Mg2 + PPorphyrin + Phytyl-PP --> AdHCYS + Chlorophyll + PPi + 2 ADP + 2 H2O + 2 Pi + 3 H + 4 NADP

Carbohydrate synthesis

R148 G1P <--> CARB + Pi

Biomass synthesis

R149 32.687 ATP + 32.687 H2O + 0.8456 PROTEIN + 0.0026 Chlorophyll + 0.0402 RNA + 0.0014 DNA + 0.1103 PA --> B + 32.687 H + 32.687 ADP + 32.687 Pi

Transport reactions

R150 # <--> CO2

R151 # <--> O2

R152 # <--> H2O

R153 # <--> Pi

R154 # <--> SO4

R155 # <--> NO3

R156 # <--> Mg2

R157	# --> Light
R158	# <--> H
R159	B --> #
160	# <--> PA
161	# <--> CARB
162	MAINT --> #

3 List of metabolites

M1	13DPG	1,3-diPhosphoglycerate
M2	2-oxobutan	2-Oxobutanoate
M3	2PG	2-Phosphoglycerate
M4	3PG	3-Phosphoglycerate
M5	5FTHF	5-Formyl-THF
M6	6PG	6-Phosphogluconate
M7	AcACP	Acetyl-ACP
M8	AcCoA	Acetyl-CoA
M9	Ace	Acetate
M10	ACP	Acetyl-carrier protein
M11	Ad	Adenosine
M12	AdHCYS	S-Adenosyl-L-homocysteine
M13	AdMET	S-Adenosyl-L-methionine
M14	ADP	Adenosine diphosphate
M15	AICAR	5-Aminoimidazole-4-carboxamide ribonucleine
M16	AKG	2-Oxoglutarate (alpha-ketoglutarate)
M17	ALA	Alanine
M18	AMP	Adenosine monophosphate
M19	ANTH	Anthranilate
M20	APS	Adenylyl sulfate
M21	ARG	Arginine
M22	ASA	L-Aspartic semialdehyde
M23	ASN	Asparagine
M24	ASP	Aspartate
M25	ATP	Adenosine triphosphate
M26	B	Functional biomass
M27	c13DPG	Chloroplast 1,3-diPhosphoglycerate
M28	C14:0ACP	Tetradecanoyl-ACP (Myristic acid)
M29	C14:1ACP	Myristoleic acid- ACP
M30	C16:0ACP	Hexadecanoyl-ACP (Palmitic acid)
M31	C16:1ACP	Trans-Hexadec-2-enoyl-ACP (Palmitoleic acid)
M32	C16:2ACP	Hexadecadienoic acid -ACP
M33	C16:3ACP	Hexadecatrienoic acid -ACP
M34	C18:0ACP	Octadecanoyl-ACP (Stearic acid)
M35	C18:1ACP	Cis-11-ocadecanoate-ACP (Oleic acid)

M36	C18:2ACP	Linoleic acid -ACP
M37	C18:3ACP	Alpha-linoleic acid -ACP
M38	C18:4ACP	Octadecatetranoic acid -ACP
M39	C20:0ACP	Arachidic acid -ACP
M40	C20:1ACP	Eicosacenoic acid -ACP
M41	C20:2ACP	Eicosadienoic acid -ACP
M42	C20:3ACP	Eicosatrienoic acid-ACP
M43	C20:4ACP	Arachodonic acid-ACP
M44	C20:5ACP	Eicosapentaenoic acid -ACP
M45	CaP	Carbamoyl phosphate
M46	CARB	Carbohydrate
M47	cDHAP	Chloroplast dihydroxyacetone
M48	CDP	Cytidine diphosphate
M49	cE4P	Chloroplast erythrose 4-phosphate
M50	cF16P	Chloroplast fructose 1,6-bisphosphate
M51	cF6P	Chloroplast fructose 6-phosphate
M52	cG3P	Chloroplast 3-phosphoglycerate
M53	cGAP	Chloroplast glyceraldehyde 3-phosphate
M54	Chlorophyll	Chlorophyll
M55	CHO	Chorismate
M56	CIT	Citrate
M57	cNADP	Chloroplast nicotinamidephosphate oxidized
M58	cNADPH	Chloroplast nicotinamidephosphate reduced
M59	CO2	Carbon dioxide
M60	CoA	Coenzyme A
M61	cR5P	Chloroplast ribose 5-phosphate
M62	cRu15DP	Chloroplast ribulose 1,5-phosphate
M63	cRu5P	Chloroplast ribulose 5-phosphate
M64	cS7P	Chloroplast sedoheptulose 7-phosphate
M65	CTP	Cytidine triphosphate
M66	cX5P	Chloroplast xylulose 5-phosphate
M67	CYS	Cysteine
M68	DAP	Diaminopimelate
M69	dATP	Deoxy ATP
M70	dCTP	Deoxy CTP
M71	dGTP	Deoxy GTP
M72	DHAP	Dihydroxyacetone-Phosphate (Glycerone-Phosphate)
M73	DHF	Dihydrofolate
M74	DNA	Deoxyribonucleic acid
M75	dTTP	Deoxy TTP
M76	E4P	Erythrose 4-phosphate
M77	F16P	Fructose 1,6-bisphosphate
M78	F6P	Fructose 6-phosphate
M79	FAD	Flavin adenine dinucleotide oxidized
M80	FADH2	Flavin adenine dinucleotide reduced

M81	FORM	Formic acid
M82	FUM	Fumarate
M83	G1P	Glucose 1-phosphate
M84	G6P	Glucose 6-phosphate
M85	GAP	Glyceraldehyde 3-phosphate
M86	GDP	Guanosine diphosphate
M87	GLN	Glutamine
M88	GLU	Glutamate
M89	GLY	Glycine
M90	GLYC3P	Glycerol 3-phosphate
M91	Glycerate	Glycerate
M92	glyoxylate	Glyoxylate
M93	GMP	Guanosine monophosphate
M94	GTP	Guanosine triphosphate
M95	H	Proton
M96	H2O	Water
M97	H2O2	Hydrogen peroxyde
M98	H2S	Hydrogen sulfur
M99	HCYS	Homocysteine
M100	HIS	Histidine
M101	HSER	Homoserine
M102	HydPro	Hydroxyproline
M103	HydPyr	3-Hydroxyproline
M104	ILE	Isoleucine
M105	IMP	Inosine monophosphate
M106	LEU	Leucine
M107	Light	Photons
M108	LYS	Lysine
M109	MAINT	Maintenance term
M110	MAL	Malate
M111	MalACP	Malonyl-ACP
M112	MalCoA	Malonyl-CoA
M113	MET	Methionine
M114	METHF	5,10-Methylene-THF
M115	Mg2	Magnesium
M116	MTHF	Methyl-THL
M117	MYLTHF	5,10-Methenyl-THF
M118	N10FTHF	10-Formyl-THF
M119	NAD	Nicotinamide oxidized
M120	NADH	Nicotinamide reduced
M121	NADP	Nicotinamidephosphate oxidized
M122	NADPH	Nicotinamidephosphate reduced
M123	NH4	Ammonium
M124	NO2	Nitrite
M125	NO3	Nitrate

M126	O2	Oxygen
M127	OXA	Oxaloacetate
M128	PA	Phosphatic Acid
M129	PEP	Phosphoenolpyruvate
M130	PHE	Phenylalanine
M131	Phytyl-PP	Phytyl-diphosphate
M132	Pi	Orthophosphate
M133	PPi	Pyrophosphate
M134	PPorphyrin	Protoporphyrine
M135	PRE	Prephanate
M136	PRO	Proline
M137	PROTEIN	Protein
M138	PRPP	Phosphorybosylpyrophosphate
M139	PYR	Pyruvate
M140	R5P	Ribose 5-phosphate
M141	RNA	Ribonucleic acid
M142	RU5P	Ribulose 5-phosphate
M143	S7P	Sedoheptulose 7-phosphate
M144	SER	Serine
M145	SO3	Sulphite
M146	SO4	Sulphate
M147	SUC	Succinate
M148	SUCCoA	Succinyl Coenzyme A
M149	THF	Tetrahydrofolate
M150	THR	Threonine
M151	TRYP	Tryptophan
M152	TYR	Tyrosine
M153	UDP	Uridine diphosphate
M154	UMP	Uridine monophosphate
M155	UTP	Uridine triphosphate
M156	VAL	Valine
M157	X5P	Xylulose 5-phosphate

4 Analysis of the whole network

Elementary flux mode analysis of the whole network yielded 18776 modes, in which 17902 yielded biomass. Flux coupling analysis, using [16], indicated than no reactions were blocked.

5 List of sub-networks

N°	Name	Reactions	Incoming metabolites	Outgoing metabolites
SN1	Photosynthesis	R1-R14	Light, Pi, CO_2, H_2O	O_2, GAP
SN2	Upper glycolysis	R16-R20	H_2O, ATP, G6P, GAP	H, ADP, Pi, G6P, GAP

SN3	Lower glycolysis	R21-R24	GAP, PEP, Pi, ATP, ADP, NADH, NAD, H_2O	GAP, PEP, Pi, ATP, ADP, NADH, NAD, H_2O
SN4	Carbohydrate synthesis	R15,R148	Pi, G6P, CARB	Pi, G6P, CARB
SN5	Lipids synthesis	R25-R26, R44, R103-R123	GAP, PEP, PA, ATP, ADP, Pi, NADH, NAD, H, NADPH, NADP, H_2O, CO_2, O_2	GAP, PEP, PA, ATP, ADP, Pi, NADH, NAD, H, NADPH, NADP, H_2O, CO_2, O_2
SN6	Biomass synthesis	R16, R21-22, R25-R43, R45-R102, R124-R147, R149	PEP, G6P, PA, H, O_2, NO_3, SO_4, Mg	H_2O, CO_2, Pi, B

6 Macroscopic reactions for biomass synthesis sub-network

6.1 Macroscopic reactions with biomass

6.1.1 List of reactions

N°	Macroscopic reaction
MR8.1	7.36572 O2 + 4.45869 H + 3.1265 PEP + 1.30971 NO3 + 1.14172 G6P + 0.1118 PA + 0.0303876 SO4 + 0.0025 Mg --> 1 B + 4.22672 Pi + 6.00312 H2O + 11.6706 CO2
MR8.2	7.36572 O2 + 4.45869 H + 3.1265 PEP + 1.30971 NO3 + 1.14172 G6P + 0.1118 PA + 0.0303876 SO4 + 0.0025 Mg --> 1 B + 4.22672 Pi + 6.00312 H2O + 11.6706 CO2
MR8.3	7.37689 O2 + 4.46315 H + 3.13097 PEP + 1.30971 NO3 + 1.14172 G6P + 0.1118 PA + 0.0303876 SO4 + 0.0025 Mg --> 1 B + 4.23119 Pi + 6.00759 H2O + 11.684 CO2
MR8.4	7.37689 O2 + 4.46315 H + 3.13097 PEP + 1.30971 NO3 + 1.14172 G6P + 0.1118 PA + 0.0303876 SO4 + 0.0025 Mg --> 1 B + 4.23119 Pi + 6.00759 H2O + 11.684 CO2
MR8.5	7.39476 O2 + 4.4703 H + 3.13812 PEP + 1.30971 NO3 + 1.14172 G6P + 0.1118 PA + 0.0303876 SO4 + 0.0025 Mg --> 1 B + 4.23834 Pi + 6.01474 H2O + 11.7055 CO2
MR8.6	7.39476 O2 + 4.4703 H + 3.13812 PEP + 1.30971 NO3 + 1.14172 G6P + 0.1118 PA + 0.0303876 SO4 + 0.0025 Mg --> 1 B + 4.23834 Pi + 6.01474 H2O + 11.7055 CO2
MR8.7	7.40593 O2 + 4.47477 H + 3.14259 PEP + 1.30971 NO3 + 1.14172 G6P + 0.1118 PA + 0.0303876 SO4 + 0.0025 Mg --> 1 B + 4.24281 Pi + 6.0192 H2O + 11.7189 CO2
MR8.8	7.40593 O2 + 4.47477 H + 3.14259 PEP + 1.30971 NO3 + 1.14172 G6P + 0.1118 PA + 0.0303876 SO4 + 0.0025 Mg --> 1 B + 4.24281 Pi + 6.0192 H2O + 11.7189 CO2
MR8.9	7.40952 O2 + 4.4762 H + 3.14402 PEP + 1.30971 NO3 + 1.14172 G6P + 0.1118 PA + 0.0303876 SO4 + 0.0025 Mg --> 1 B + 4.24424 Pi + 6.02064 H2O + 11.7232 CO2
MR8.10	7.40952 O2 + 4.4762 H + 3.14402 PEP + 1.30971 NO3 + 1.14172 G6P + 0.1118 PA + 0.0303876 SO4 + 0.0025 Mg --> 1 B + 4.24424 Pi + 6.02064 H2O + 11.7232 CO2
MR8.11	7.42069 O2 + 4.48067 H + 3.14849 PEP + 1.30971 NO3 + 1.14172 G6P + 0.1118 PA + 0.0303876 SO4 + 0.0025 Mg --> 1 B + 4.24871 Pi + 6.02511 H2O + 11.7366 CO2
MR8.12	7.42069 O2 + 4.48067 H + 3.14849 PEP + 1.30971 NO3 + 1.14172 G6P + 0.1118 PA + 0.0303876 SO4 + 0.0025 Mg --> 1 B + 4.24871 Pi + 6.02511 H2O + 11.7366 CO2
MR8.13	7.43856 O2 + 4.48782 H + 3.15564 PEP + 1.30971 NO3 + 1.14172 G6P + 0.1118 PA + 0.0303876 SO4 + 0.0025 Mg --> 1 B + 4.25586 Pi + 6.03225 H2O + 11.758 CO2
MR8.14	7.43856 O2 + 4.48782 H + 3.15564 PEP + 1.30971 NO3 + 1.14172 G6P + 0.1118 PA + 0.0303876 SO4 + 0.0025 Mg --> 1 B + 4.25586 Pi + 6.03225 H2O + 11.758 CO2
MR8.15	7.44973 O2 + 4.49229 H + 3.16011 PEP + 1.30971 NO3 + 1.14172 G6P + 0.1118 PA +

	0.0303876 SO4 + 0.0025 Mg --> 1 B+ 4.26033 Pi + 6.03672 H2O + 11.7714 CO2
MR8.16	7.44973 O2 + 4.49229 H + 3.16011 PEP + 1.30971 NO3 + 1.14172 G6P + 0.1118 PA + 0.0303876 SO4 + 0.0025 Mg2 --> 1 B + 4.26033 Pi + 6.03672 H2O + 11.7714 CO2
MR8.17	7.80512 O2 + 4.63445 H + 3.30226 PEP + 1.30971 NO3 + 1.14172 G6P + 0.1118 PA + 0.0303876 SO4 + 0.0025 Mg --> 1 B + 4.40248 Pi + 6.17888 H2O + 12.1979 CO2
MR8.18	7.80512 O2 + 4.63445 H + 3.30226 PEP + 1.30971 NO3 + 1.14172 G6P + 0.1118 PA + 0.0303876 SO4 + 0.0025 Mg --> 1 B + 4.40248 Pi + 6.17888 H2O + 12.1979 CO2
MR8.19	7.81629 O2 + 4.63891 H + 3.30673 PEP + 1.30971 NO3 + 1.14172 G6P + 0.1118 PA + 0.0303876 SO4 + 0.0025 Mg --> 1 B + 4.40695 Pi + 6.18335 H2O + 12.2113 CO2
MR8.20	7.81629 O2 + 4.63891 H + 3.30673 PEP + 1.30971 NO3 + 1.14172 G6P + 0.1118 PA + 0.0303876 SO4 + 0.0025 Mg --> 1 B + 4.40695 Pi + 6.18335 H2O + 12.2113 CO2
MR8.21	7.83416 O2 + 4.64606 H + 3.31388 PEP + 1.30971 NO3 + 1.14172 G6P + 0.1118 PA + 0.0303876 SO4 + 0.0025 Mg --> 1 B + 4.4141 Pi + 6.1905 H2O + 12.2328 CO2
MR8.22	7.83416 O2 + 4.64606 H + 3.31388 PEP + 1.30971 NO3 + 1.14172 G6P + 0.1118 PA + 0.0303876 SO4 + 0.0025 Mg --> 1 B + 4.4141 Pi + 6.1905 H2O + 12.2328 CO2
MR8.23	7.84533 O2 + 4.65053 H + 3.31835 PEP + 1.30971 NO3 + 1.14172 G6P + 0.1118 PA + 0.0303876 SO4 + 0.0025 Mg --> 1 B + 4.41857 Pi + 6.19496 H2O + 12.2462 CO2
MR8.24	7.84533 O2 + 4.65053 H + 3.31835 PEP + 1.30971 NO3 + 1.14172 G6P + 0.1118 PA + 0.0303876 SO4 + 0.0025 Mg --> 1 B + 4.41857 Pi + 6.19496 H2O + 12.2462 CO2

6.1.2 Principal component analysis

We projected the elementary flux modes normalized by unit of functional biomass into the yield space (PEP function of CO_2). There seemed to be two type of metabolic behavior: low production and high production of CO_2 (Figure S1).

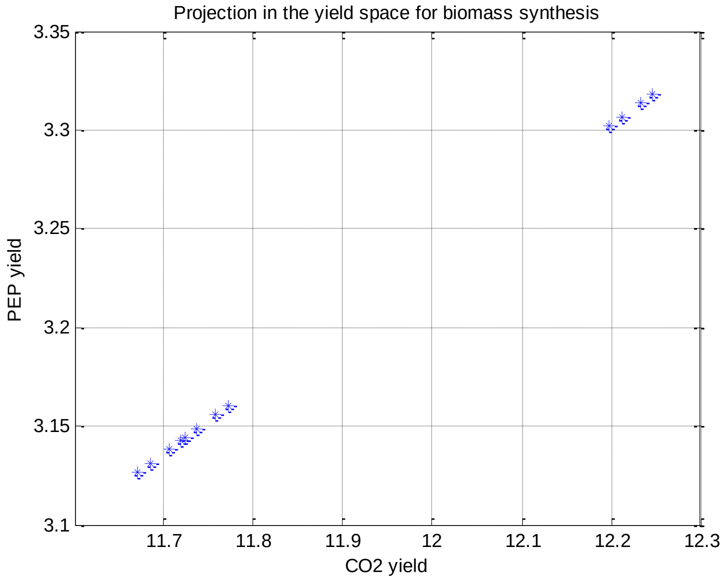

Figure S1: Projection in the yield space of elementary flux modes obtained from the biomass synthesis sub-network.

To understand the differences between these two groups, we performed a principal component analysis on the elementary flux modes (Figure S2).

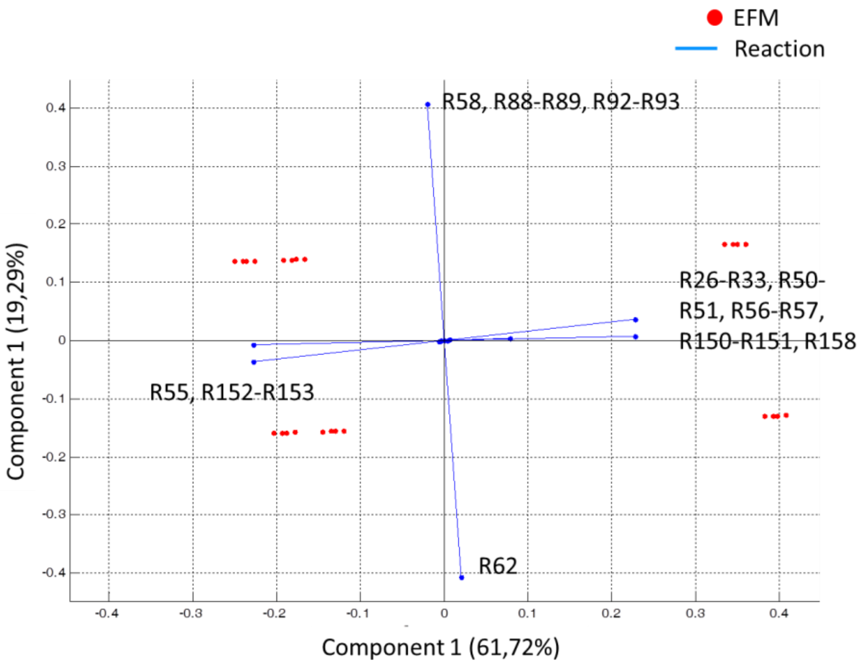

Figure S2: Principal component analysis of the elementary flux modes obtained from the biomass synthesis sub-network. The first two axes bear 80.81% of the information.

For the first component (61.72% of information data), CO_2 excretion (R150) and O_2 consumption (R151) are correlated with PEP consumption through the citric acid cycle (R26-R33) and oxidative phosphorylation (R50-R51). Indeed, if more PEP is used, the citric acid cycle is more used and thus CO_2 excretion and O_2 consumption increase. The difference of PEP consumption is due to the way nitrogen is incorporated: either with glutamine (R55) or with glutamate (R56-R57). Incorporation of azote with glutamate is energetically less efficient that with glutamine. Indeed, nitrogen incorporation with glutamine only requires NADPH whereas incorporation with glutamate requires NADPH and ATP, hence oxidative phosphorylation for ATP generation.

For the second component (19.29% of data information), EFM are different mainly because of the way alanine is synthesized. Indeed, alanine is either synthesized directly from glutamine and pyruvate (R62), or from glyoxylate (R58,R88-R89,R92-93).

6.2 Macroscopic reactions with no biomass

N°	Macroscopic reaction	Reactions taking place	Explanation
MR8.25	2.5 O2 + 1 PEP + 1 H --> 1 H2O + 1 Pi + 3 CO2	R25-R35, R50-R51	Citric acid cycle + oxidative phosphorylation + anaplerotic reactions
MR8.26	2.5 O2 + 1 H + 1 PEP --> 1 Pi + 1 H2O + 3 CO2	R25-R33, R35-R36, R50-R51	Citric acid cycle + oxidative phosphorylation

			+ anaplerotic reactions
MR8.27	2.5 O2 + 1 H + 1 PEP --> 1 Pi + 1 H2O + 3 CO2	R25-R33, R50-R51, R54	Citric acid cycle + oxidative phosphorylation + maintenance
MR8.28	2.5 O2 + 1 H + 1 PEP --> 1 Pi + 1 H2O + 3 CO2	R25-R33, R50-R51, R97, R101	Citric acid cycle + oxidative phosphorylation + THF metabolism
MR8.29	2.5 O2 + 1 H + 1 PEP --> 1 Pi + 1 H2O + 3 CO2	R25-R33, R50-R51, R95-R96, R100	Citric acid cycle + oxidative phosphorylation + THF metabolism
MR8.30	-	R25, R34, R36	Anaplerotic reactions

7 Metabolic engineering: deletion of MR6 forward reaction (simulation conditions)

We tested the effect of the suppression of MR6 forward reaction on the metabolites concentration of the model. The two models were then simulated for 48h, one with $k_{carb} = 0\ \mathrm{h}^{-1}.\mathrm{mM\ B}^{-1}$, the other one with $k_{carb} = 70.00\ \mathrm{h}^{-1}.\mathrm{mM\ B}^{-1}$. The dilution rate and the incoming substrate concentrations were set at 1 days^{-1} and 4.018 mgN.L^{-1}.

References

1. Kliphuis AMJ, Klok AJ, Martens DE, Lamers PP, Janssen M, et al. (2011) Metabolic modeling of Chlamydomonas reinhardtii: energy requirements for photoautotrophic growth and maintenance. J Appl Phycol 24: 253–266.

2. Yang C, Hua Q, Shimizu K (2000) Energetics and carbon metabolism during growth of microalgal cells under photoautotrophic, mixotrophic and cyclic light-autotrophic/dark-heterotrophic conditions. Biochem Eng J 6: 87–102.

3. Boyle NR, Morgan JA (2009) Flux balance analysis of primary metabolism in Chlamydomonas reinhardtii. BMC Syst Biol 3: 1–14.

4. Manichaikul A, Ghamsari L, Hom E, Chin C, Murray R, et al. (2009) Metabolic network analysis integrated with transcript verification for sequenced genomes. Nat Methods 6: 589–592.

5. Chang RL, Ghamsari L, Manichaikul A, Hom EFY, Balaji S, et al. (2011) Metabolic network reconstruction of Chlamydomonas offers insight into light-driven algal metabolism. Mol Syst Biol 7: 1–13.

6. Dal'Molin CGDO, Quek L-E, Palfreyman RW, Nielsen LK (2011) AlgaGEM--a genome-scale metabolic reconstruction of algae based on the Chlamydomonas reinhardtii genome. BMC Genomics 12 Suppl 4: S5.

7. Cogne G, Rügen M, Bockmayr A, Titica M, Dussap C-G, et al. (2011) A model-based method for investigating bioenergetic processes in autotrophically growing eukaryotic microalgae: application to the green algae Chlamydomonas reinhardtii. Biotechnol Prog 27: 631–640.

8. Krumholz EW, Yang H, Weisenhorn P, Henry CS, Libourel IGL (2012) Genome-wide metabolic network reconstruction of the picoalga Ostreococcus. J Exp Bot 63: 2353–2362.

9. Lacour T, Sciandra A, Talec A, Mayzaud P, Bernard O (2012) Diel Variations of Carbohydrates and Neutral Lipids in Nitrogen-Sufficient and Nitrogen-Starved Cyclostat Cultures of Isochrysis Sp. J Phycol 48: 966–975.

10. Griffiths MJ, Hille RP, Harrison STL (2011) Lipid productivity, settling potential and fatty acid profile of 11 microalgal species grown under nitrogen replete and limited conditions. J Appl Phycol 24: 989–1001.

11. Brown MR (1991) The amino-acid and sugar composition of 16 species of microalgae used in mariculture. J Exp Mar Bio Ecol 145: 79–99.

12. Geider R, La Roche J (2002) Redfield revisited: variability of C:N:P in marine microalgae and its biochemical basis. Eur J Phycol 37: 1–17.

13. MacIntyre H, Kana T, Anning T (2002) Photoacclimation of Photosynthesis Irradiance Response Curves and Photosynthetic Pigments in Microalgae and Cyanobacteria1. J Phycol 38: 17–38.

14. Cheung CYM, Williams TCR, Poolman MG, Fell D a., Ratcliffe RG, et al. (2013) A method for accounting for maintenance costs in flux balance analysis improves the prediction of plant cell metabolic phenotypes under stress conditions. Plant J 75: 1050–1061.

15. Young JD, Shastri AA, Stephanopoulos G, Morgan JA (2011) Mapping photoautotrophic metabolism with isotopically nonstationary 13 C flux analysis. Metab Eng 13: 656–665.

16. Larhlimi A, David L, Selbig J, Bockmayr A (2012) F2C2: a fast tool for the computation of flux coupling in genome-scale metabolic networks. BMC Bioinformatics 13: 1–9.
